# Bacterial Long-Range Warfare: Aerial Killing of Legionella pneumophila by Pseudomonas fluorescens

**DOI:** 10.1128/spectrum.00404-21

**Published:** 2021-08-11

**Authors:** Marie-Hélène Corre, Anne Mercier, Mathilde Bouteiller, Alix Khalil, Christophe Ginevra, Ségolène Depayras, Charly Dupont, Meg Rouxel, Mathias Gallique, Laettitia Grac, Sophie Jarraud, David Giron, Annabelle Merieau, Jean-Marc Berjeaud, Julien Verdon

**Affiliations:** a Laboratoire Ecologie & Biologie des Interactions, UMR CNRS 7267, Université de Poitiers, Poitiers, France; b Laboratoire de Microbiologie Signaux et Microenvironnement, EA 4312, Université de Rouen, Rouen, France; c Institut de Recherche sur la Biologie de l’Insecte (IRBI), UMR 7261 CNRS/Université de Tours, Tours, France; d Laboratoire de Génie des Procédés, Environnement, Agroalimentaire, UMR CNRS 6144, Oniris Ecole Nationale Vétérinaire, Agroalimentaire et de l’Alimentation, Nantes, France; e Centre National de Référence des Légionelles, Institut des Agents Infectieux, Hospices Civils de Lyon, Lyon, France; f CIRI, Centre International de Recherche en Infectiologie, Inserm, U1111, CNRS, UMR 5308, Université Lyon 1, École Normale Supérieure de Lyon, Lyon, France; g Meakins-Christie laboratories, Research Institute of the McGill University Health Centre, Montreal, Quebec, Canada; h Department of Chemical Engineering, McGill University, Montreal, Quebec, Canada; Emory University School of Medicine

**Keywords:** *Legionella*, *Pseudomonas*, volatile organic compounds, 1-undecene, antibacterial activity, SPME

## Abstract

Legionella pneumophila, the causative agent of Legionnaires’ disease, is mostly found in man-made water systems and is one of the most closely monitored waterborne pathogens. With the aim of finding natural ways to control waterborne pathogens and thus further reduce the impact of disinfection by-products on human health, some studies have demonstrated the ability of bacteria to kill *Legionella* through the production of secondary metabolites or antimicrobial compounds. Here, we describe an unexpected growth inhibition of L. pneumophila when exposed to a physically separated strain of Pseudomonas fluorescens, designated as MFE01. Most of the members of the *Legionellaceae* family are sensitive to the volatile substances emitted by MFE01, unlike other bacteria tested. Using headspace solid-phase microextraction GC-MS strategy, a volatilome comparison revealed that emission of 1-undecene, 2-undecanone, and 2-tridecanone were mainly reduced in a Tn*5*-transposon mutant unable to inhibit at distance the growth of L. pneumophila strain Lens. We showed that 1-undecene was mainly responsible for the inhibition at distance *in vitro*, and led to cell lysis in small amounts, as determined by gas chromatography-mass spectrometry (GC-MS). Collectively, our results provide new insights into the mode of action of bacterial volatiles and highlight them as potent anti-*Legionella* agents to focus research on novel strategies to fight legionellosis.

**IMPORTANCE** Microbial volatile compounds are molecules whose activities are increasingly attracting the attention of researchers. Indeed, they can act as key compounds in long-distance intrakingdom and interkingdom communication, but also as antimicrobials in competition and predation. In fact, most studies to date have focused on their antifungal activities and only a few have reported on their antibacterial properties. Here, we describe that 1-undecene, naturally produced by P. fluorescens, is a volatile with potent activity against bacteria of the genus *Legionella*. In small amounts, it is capable of inducing cell lysis even when the producing strain is physically separated from the target. This is the first time that such activity is described. This molecule could therefore constitute an efficient compound to counter bacterial pathogens whose treatment may fail, particularly in pulmonary diseases. Indeed, inhalation of these volatiles should be considered as a possible route of therapy in addition to antibiotic treatment.

## INTRODUCTION

Legionella pneumophila is the main causative agent of Legionnaires’ disease (LD), a serious and life-threatening form of pneumonia. Specifically, the serogroup 1 is associated with 82.9% of LD cases in Europe (1, 2) and more than 80% of cases worldwide (3, 4). This bacterium originates from freshwater environments and colonizes engineered water systems, where it can persist within multispecies biofilms and phagotrophic protists such as free-living amoebae (5). Indeed, this opportunistic pathogen is able to resist phagocytic predation and replicate inside this nutrient-rich protective environment in a dedicated compartment called the *Legionella*-containing vacuole (6). Inhalation of contaminated aerosols is the main route of exposure in humans, although one case of person-to-person transmission has recently been documented (7, 8). In many water settings (e.g., drinking water supplies, hot water systems, and cooling towers), *Legionella* spp. represent a particular concern and remain one of the most closely monitored microbial agents (9). Multiplication of *Legionella* spp. in those artificial water systems is highly facilitated by some abiotic factors such as warm temperatures and water stagnation (10). Inadequate maintenance or lack of water disinfection processes in these systems also contributes to their persistence. Moreover, microbial inhabitants play a key role in the survival of L. pneumophila, notably through the grazing of free-living amoebae on biofilms and interactions with other bacteria (11, 12). Indeed, some bacteria were shown to promote L. pneumophila survival in oligotrophic environments, while others were revealed to antagonize its persistence within biofilms (5, 13). Furthermore, we have recently highlighted that more than 60% of freshwater cultivable bacterial isolates exhibit antagonistic activity against L. pneumophila (14). Of these isolates with anti-*Legionella* activity, 77% belong to the genera Pseudomonas, *Bacillus*, *Flavobacterium*, or *Aeromonas*. Pseudomonas isolates were of particular interest as we have shown that anti-*Legionella* molecules included both diffusible secreted compounds and volatile organic compounds (VOCs). A part of the secreted molecules has been previously characterized and corresponds to lipopeptides and rhamnolipids (15). As VOCs are hydrophobic compounds, this *Legionella*/Pseudomonas interaction is more likely to occur in soil than in water. However, characterization of the effect of VOC inhibition on L. pneumophila has not been explored.

VOCs are a large group of secondary metabolites produced by many lifeforms on earth, such as humans, animals, plants, fungi, and bacteria (16, 17). Since human olfaction can sense thousands of volatiles molecules, VOCs have been exploited for a long time in several different areas (e.g., production of fermented products or creation of perfumes). Although it is unclear how microorganisms can sense those compounds, microbial VOCs were shown to play key roles in microbial interactions between physically separated microorganisms (18). To date, soil and rhizosphere environments remain the best-studied systems for the production and activities of microbial VOCs, particularly with regard to plant-bacteria and bacteria-fungi interactions (18, 19). Relationships between bacteria were also explored and many positive and negative effects were characterized for those bacterial VOCs. Indeed, they can modulate the antibiotic tolerance pattern of bacterial cells (20–22), increase bacterial motility (22, 23), enhance bacterial capacity to form and to disperse biofilms (22, 24–26), and modulate bacterial virulence (27, 28). Conversely, bacterial VOCs can also inhibit biofilm formation (22, 29, 30), impair cell motility (21), and exert direct antagonistic effects for microbial neighborhoods (29, 31–33). While volatile-mediated antifungal activity is well described in the literature, very few studies have reported data concerning volatile-mediated antibacterial activity (34).

We used P. fluorescens strain MFE01 as model, since bacteria of the same species have been previously reported to inhibit the growth of L. pneumophila (14). In this perspective, we identified the main VOCs of this strain and unraveled a part of their action on L. pneumophila. A two-petri-dish assay was designed to experimentally screen numerous bacterial strains to determine the activity spectrum of VOCs produced by the P. fluorescens MFE01 strain. The MFE01 strain volatilome was then characterized using headspace solid-phase microextraction (SPME) coupled with gas chromatography-mass spectrometry (GC-MS). Mutants were generated to identify VOCs involved in anti-*Legionella* activity and to unravel the metabolic pathways leading to their production.

## RESULTS

### Volatiles emitted by the Pseudomonas fluorescens MFE01 strain inhibit the growth of *Legionella* species at a distance.

**FIG 1:**
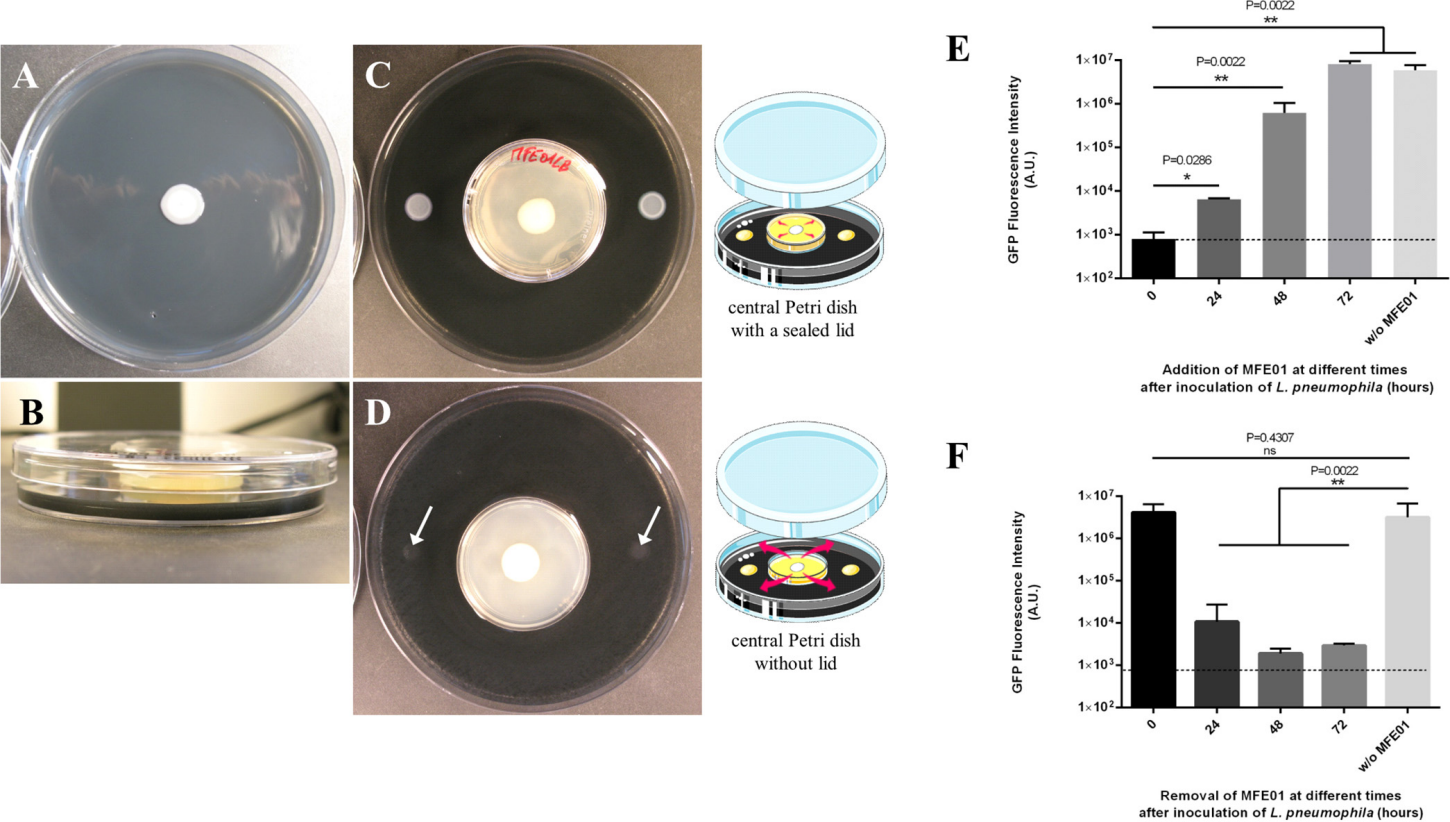
Antagonistic activity of P. fluorescens MFE01 toward L. pneumophila Lens. (A) Spot-on-lawn assay with P. fluorescens MFE01 (central spot). (B) Side view of the experimental two-petri-dish assay. (C) P. fluorescens MFE01 grown in a central small petri dish with a lid sealed with parafilm. (D) P. fluorescens MFE01 grown in a central small petri dish without lid. Growth of L. pneumophila Lens was monitored after 96 h of incubation at 28°C. Deposits of *L. pneumophila* on the agar are indicated by white arrows. The absence of bacterial growth indicates a volatile-dependent inhibitory phenotype. The pictures are representative of more than 6 experiments. (E) Ability of P. fluorescens MFE01 to stop at distance the growth of L. pneumophila Lens. MFE01 was spread on the center of a 6-well plate at different times after L. pneumophila was spread on the upper two sides of the 6-well plate. Fluorescence was quantified using a GFP-tagged L. pneumophila Lens in presence and absence of MFE01. The dash line represents the fluorescence intensity quantified for the reference control corresponding to the simultaneous spread of the two strains (at *t* = 0 h). **, *P < *0.01; *, *P < *0.05, compared to the condition “*t* = 0 h” (Mann-Whitney U test). Data represent the mean (± standard deviation, SD) of three independent experiments, each performed in duplicate. Error bars indicate SDs. A.U. indicates arbitrary units. (F) Irreversible inhibition of L. pneumophila Lens by P. fluorescens MFE01. MFE01 was removed at different times by cutting the agar with a scalpel blade and placing the piece in a new plate at 37°C for 96 h before GFP quantification. The dash line represents the fluorescence intensity quantified for the reference control corresponding to the simultaneous spread of the two strains (at *t* = 0 h); w/o MFE01, test without MFE01. **, *P < *0.01, compared to the condition “w/o MFE01” (Mann-Whitney U test). A *P* value > 0.05 was considered to be statistically nonsignificant (ns). Data represent the mean (± SD) of three independent experiments, each performed in duplicate. Error bars indicate SDs. A.U. indicates arbitrary units.

The P. fluorescens MFE01 strain was found to inhibit the growth of L. pneumophila strain Lens using the spot-on-lawn method (Fig. 1A). This strain displayed a full inhibition profile, as no *Legionella* colony was detected on the entire petri dish. This observation suggests that at least one highly diffusible and/or volatile antagonistic molecule is produced by MFE01. To get an in-depth understanding, a two-petri-dish assay (Fig. 1B to D), adapted from a previous study (20), was used. MFE01 was physically separated from L. pneumophila by a small petri dish sealed or not with a lid and the activity of bacterial volatiles was qualitatively estimated by the presence/absence of L. pneumophila colonies. When MFE01 was grown inside the sealed small petri dish, L. pneumophila was able to form colonies as expected (Fig. 1C). Conversely, the absence of a lid during the culture of MFE01 led to the inhibition of L. pneumophila growth (Fig. 1D). This result clearly indicates a *Legionella* volatile-dependent inhibitory phenotype driven by at least one volatile compound emitted by P. fluorescens MFE01.

To investigate whether the volatile molecule(s) emitted by P. fluorescens MFE01 could affect the growth of other bacteria, several *Legionella* and non-*Legionella* strains were cultivated in the presence of MFE01 (Table 1). Almost all *Legionella* strains tested were sensitive with the exception of L. pneumophila strain Chicago 8 (serogroup 7) and strains of Legionella feelei. The inhibition was not total for some *Legionella* species, although the growth was dramatically reduced. In contrast, the growth of all non-*Legionella* species tested in our study was never inhibited. Taken together, these results highlight a wide sensitivity of the genus *Legionella* to the action of volatile compound(s) emitted by P. fluorescens MFE01.

**TABLE 1:** Inhibitory activity of volatile compound(s) emitted by P. fluorescens MFE01

Target species				Growth in presence of MFE01^*b*^
Designation		Collection/reference^*a*^	Origin	
Legionella pneumophila ^*c*^				
* Lp* Sg1	Lens	CIP 108286	clinical	0
* Lp* Sg1	Lorraine	CIP 108729	clinical	0
* Lp* Sg1	Paris	CIP 107629	clinical	+
* Lp* Sg1	ST701	CNR 018065250501	clinical	0
* Lp* Sg1	ST435	CNR 018066068201	clinical	0
* Lp* Sg1	ST2692	CNR 018057879501	clinical	0
* Lp* Sg1	ST1	CNR 018114081801	environmental	0
* Lp* Sg1	ST13	CNR 018171015001	environmental	0
* Lp* Sg1	ST23	CNR 018206934501	environmental	0
* Lp* Sg1	ST23	CNR 018203101701	environmental	0
* Lp* Sg1	ST107	CNR 018124749101	environmental	0
* Lp* Sg1	ST146	CNR 018200116901	environmental	0
* Lp* Sg1	ST733	CNR 018080098401	environmental	0
* Lp* Sg1	ST560	CNR 018171031501	environmental	0
* Lp* Sg1	ST2721	CNR 018118808601	environmental	0
* Lp* Sg2	Togus-1	ATCC 33154	clinical	0
* Lp* Sg3	Bloomington-2	ATCC 33155	environmental	0
* Lp* Sg4	Los Angeles-1	ATCC 33156	clinical	0
* Lp* Sg5	Dallas 1E	ATCC 33216	environmental	+
* Lp* Sg6	Chicago 2	ATCC 33215	clinical	0
* Lp* Sg7	ST39	CNR 019076187101	clinical	0
* Lp* Sg7	ST1904	CNR 015197545401	clinical	0
* Lp* Sg7	ST2116	CNR 015173672801	clinical	0
* Lp* Sg7	Chicago 8	ATCC 33823	environmental	++
* Lp* Sg8	Concord 3	ATCC 35096	clinical	0
* Lp* Sg8	ST1324	CNR 018175859001	environmental	0
* Lp* Sg9	IN-23-G1-C2	ATCC 35289	environmental	+
* Lp* Sg10	Leiden 1	ATCC 43283	clinical	0
* Lp* Sg11	797-PA-H	ATCC 43130	clinical	0
* Lp* Sg12	570-CO-H	ATCC 43290	clinical	0
* Lp* Sg13	82A3105	ATCC 43736	clinical	+
* Lp* Sg14	1169-MN-H	ATCC 43703	clinical	0
* Lp* Sg15	Lansing 3	ATCC 35251	clinical	0
*Legionella* spp.				
* L. bozemanae* WIGA		ATCC 33217	clinical	0
* L. dumoffii* NY 23		ATCC 33279	environmental	+
* L. feeleii*		CNR 017092681801	clinical	++
* L. feeleii* WO-44C		ATCC 35072	environmental	++
* L. feeleii*		CNR 018035518201	environmental	++
* L. feeleii*		CNR 018054338601	environmental	++
* L. longbeachae* Tucker 1		ATCC 33484	clinical	0
* L. micdadei* TATLOCK		ATCC 33218	clinical	0
* L. oakridgensis* Oak Ridge 10		ATCC 33761	environmental	0
Other species				
* *Gram-negative bacteria				
* *Enterobacter cloacae D03		Laboratory collection	environmental	++
* * Escherichia coli		LMG 2092	clinical	++
* *Klebsiella pneumoniae 0502083		Laboratory collection	clinical	++
* *Proteus mirabilis Hauser		ATCC 35659	-^*d*^	++
* *Salmonella enterica J18		Laboratory collection	clinical	++
Gram-positive bacteria				
* *Bacillus megaterium F04		Laboratory collection	environmental	++
* *Bacillus subtilis AM1		LMG 28342	environmental	++
* *Enterococcus faecalis V583		ATCC 700802	clinical	++
* *Listeria ivanovii Li4pVS2		Laboratory collection	environmental	++
* * Micrococcus luteus		ATCC 4698	environmental	++
* *Mycobacterium llatzerense EDP_4		Laboratory collection	environmental	++
* *Staphylococcus aureus Wichita		ATCC 29213	clinical	++
* *Staphylococcus warneri RK		Laboratory collection	environmental	++

a Bacterial strains were obtained from various culture collections: ATCC, American Type Culture Collection; CIP, Collection Institut Pasteur, France; CNR Légionelles, Centre National de Référence des Légionelles, Lyon, France; BCCM/LMG, Belgian Coordinated Collections of Microorganisms/Laboratory of Microbiology, Department of Biochemistry and Microbiology, Faculty of Sciences of Ghent University, Belgium. Other strains were from the laboratory culture collection.

b 0, no growth was observed; ++, growth was identical to that of the control in the absence of MFE01; +, visible growth but bacterial density was lower than that of the control.

c Sg, serogroup, ST, sequence type.

d Not provided.

### Growth of L. pneumophila Lens is irreversibly inhibited.

To support the long-range inhibition of L. pneumophila, we designed a 6-well plate inhibition assay and used a green fluorescent protein (GFP)-expressing L. pneumophila Lens strain to quantitatively measure the bacterial development. First, P. fluorescens MFE01 was added after previously inoculating L. pneumophila either at the same time or 24, 48, or 72 h later. Results showed the growth of L. pneumophila Lens was further inhibited by the early addition of MFE01, especially in the range of 0 to 48 h after inoculation of the target strain (Fig. 1E). In a second step, both strains were inoculated at the same time before removal of MFE01 from the plates after 24, 48, or 72 h of coincubation. The plates were then left at 37°C for 96 h after removal of MFE01. Regardless of the condition, the growth of L. pneumophila was never restored (Fig. 1F). Overall, the aerial exposure to volatile molecule(s) released at distance by P. fluorescens MFE01 appears lethal for L. pneumophila Lens, especially when this exposure occurs during the early stages of bacterial development.

**FIG 2:**
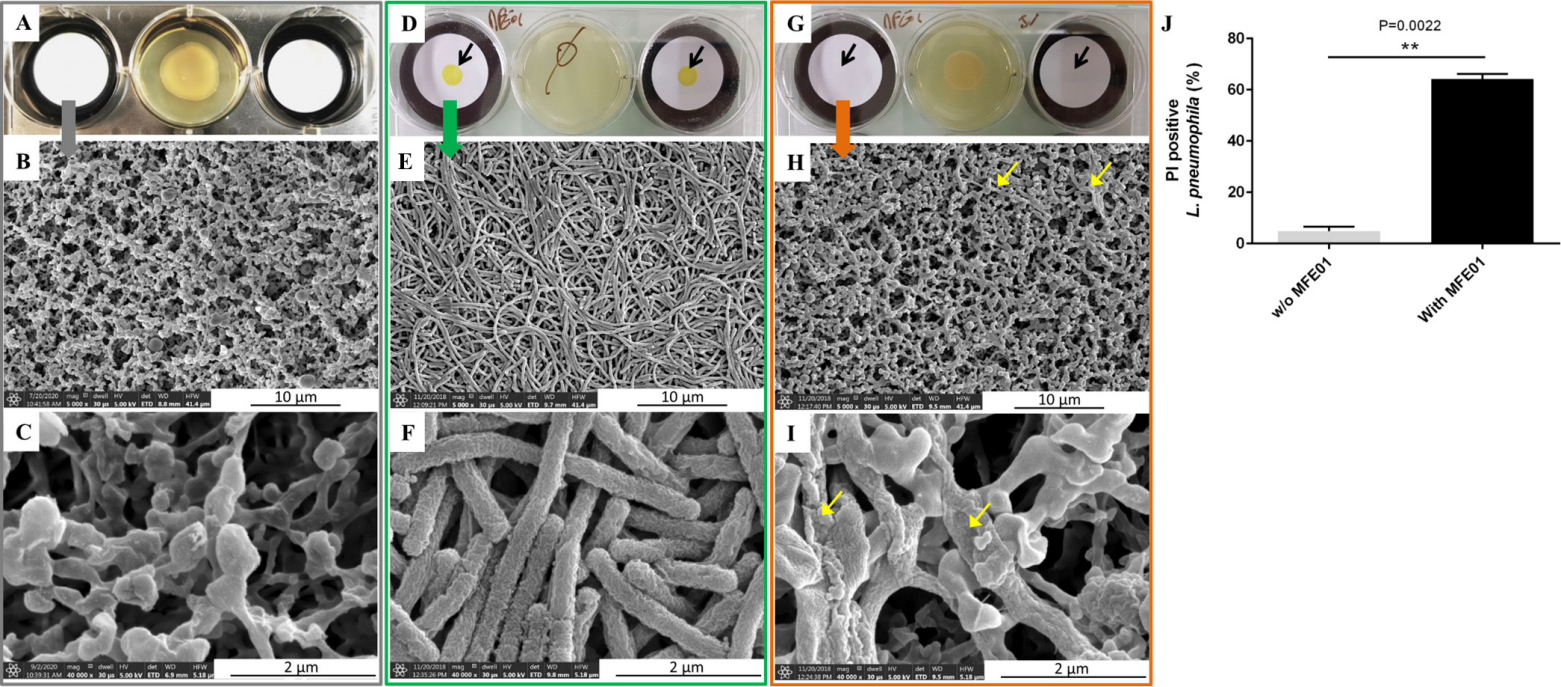
Lytic activity of L. pneumophila Lens induced by the release of VOCs from P. fluorescens MFE01. GFP-tagged L. pneumophila Lens was grown in a 6-well plate on a nitrocellulose filter (0.22 μm) at 28°C for 72 h. The initial plating of the bacterial suspension on the agar plate is indicated by black arrows. Then, filters were collected and immersed in 2.5% glutaraldehyde for fixation before scanning electron microscopy analysis. Each colored rectangle contains a distinct treatment condition with the activity test and the associated nitrocellulose filters observed in SEM. The central well may or may not contain a culture of MFE01 and the wells on either side contain either nitrocellulose filters alone or nitrocellulose filters with L. pneumophila Lens deposited on them. (A to C) Scanning electron micrographs of filters alone exposed to physically separated P. fluorescens MFE01 spread on LB agar. (D to F) Control condition with only the presence of L. pneumophila Lens. (G to I) L. pneumophila grown in the presence of physically separated P. fluorescens MFE01 spread on LB agar. Damaged bacilli are indicated by yellow arrows. Pictures are representative of four independent assays. (J) Envelope permeabilization of GFP-tagged L. pneumophila Lens. Bacteria were harvested from nitrocellulose filter (0.22 μm), labeled 15 min with propidium iodide (PI), and were subjected to flow cytometry analysis. w/o MFE01, test without MFE01; **, *P < *0.01, compared to permeabilization in the absence of MFE01 (Mann-Whitney U test). Data represent the mean (± SD) of three independent experiments, each performed in duplicate. Error bars indicate SDs.

To gain insight into the direct effect of VOCs on aerially exposed L. pneumophila cells, a scanning electron microscopy (SEM) approach was used. L. pneumophila cells were deposited on a nitrocellulose filter to grow, then exposed or not to VOCs before sampling (Fig. 2A, D, and G). In the control condition, numerous elongated bacilli crowded on top of each other were observed (Fig. 2E). A zoom highlights the membrane integrity conservation of most bacteria (Fig. 2F). When exposed to MFE01 during 72 h, only a few bacilli scattered on the filter were observable and only at higher magnification (Fig. 2H and I) in comparison with the filter alone (Fig. 2B and C). In addition, these bacilli were deformed, have lost their overall shape and appeared to be emptied of their cytoplasmic content (Fig. 2I). In order to demonstrate this lysis, the permeability of the bacterial envelope was assayed under the same conditions using propidium iodide (PI) (Fig. 2J). When exposed to MFE01, very few L. pneumophila were recovered from the filter after 72 h of exposure, unlike the unexposed control. Nevertheless, more than 60% of the population recovered from the filter was labeled with PI, which confirms that at least a portion of the population no longer had an integral envelope. All of these data illustrate the damaging activity of the VOCs emitted by MFE01 and reveal an unexpected lytic effect on L. pneumophila.

### Decreased production of volatiles in P. fluorescens MFE01-3H5 mutant compromises aerial inhibition of L. pneumophila growth.

**FIG 3:**
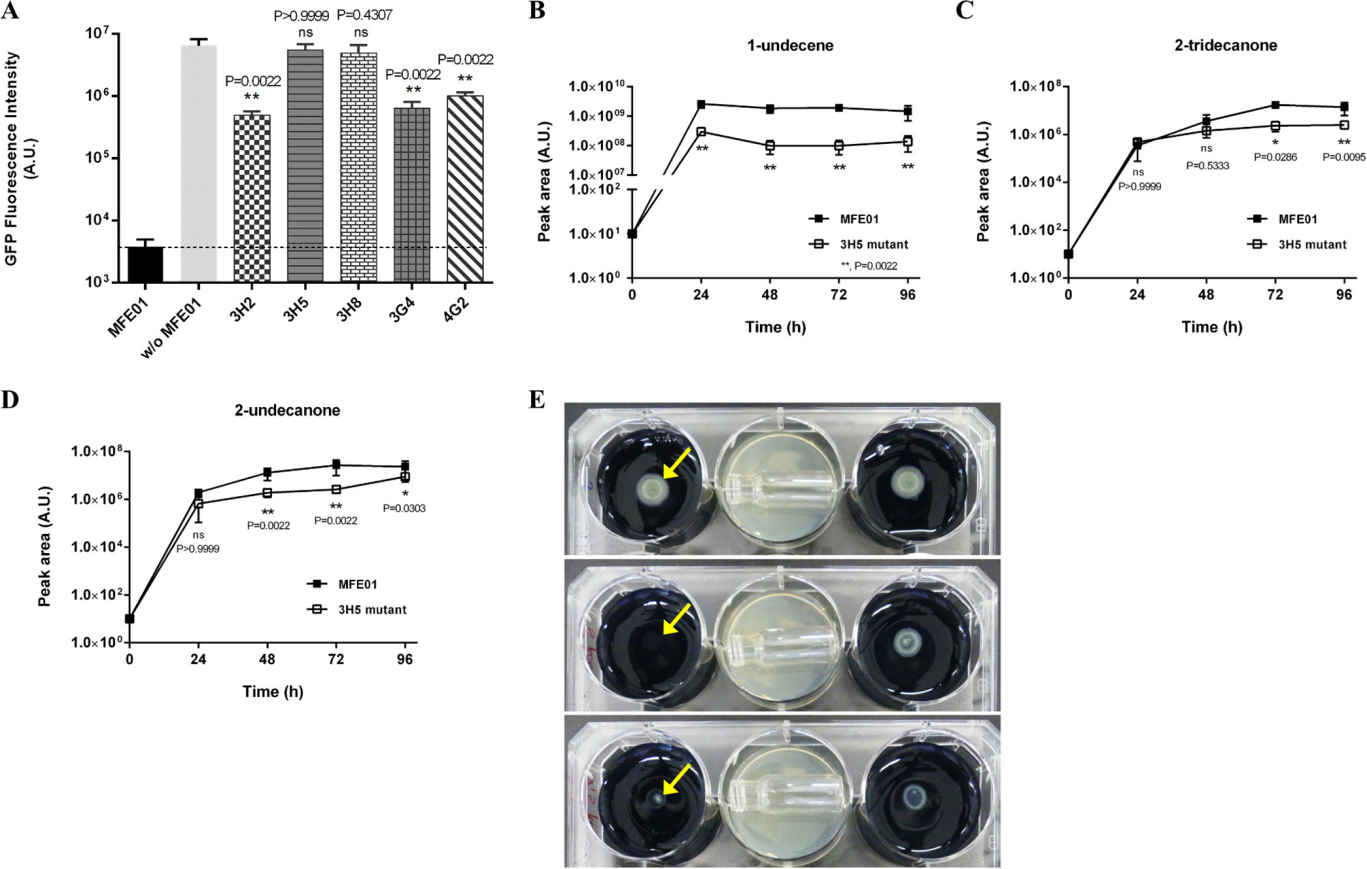
Screening of MFE01 mutants and volatiles production. (A) Long-distance inhibition of GFP-tagged L. pneumophila Lens through the production of volatile compound(s) produced by P. fluorescens MFE01 transposition mutants (named 3H2, 3H5, 3H8, 3G4, and 4G2) using the 6-well plate assay. w/o MFE01, test without MFE01; **, *P < *0.01, compared to the condition “w/o MFE01” (Mann-Whitney U test) (*n* = 6). (B to D) SPME/GC-MS kinetics of 1-undecene (B), 2-undecanone (D), and 2-tridecanone (C) produced by P. fluorescens MFE01 and its 3H5 mutant. Each strain was cultured in LB medium for 24 h and a suspension diluted at OD_600_ = 1 was deposited on a sterile GC vial filled with LB agar. Vials were then incubated at 28°C and analyzed at different time points by SPME/GC-MS. **, *P < *0.01; *, *P < *0.05, compared to the peak area obtained with MFE01 (Mann-Whitney U test). A *P* value of 0.05 was considered to be statistically nonsignificant (ns). Data represent the mean (± SD) of three independent experiments, each performed in duplicate. Error bars indicate SDs. A.U. indicates arbitrary units. (E) Long-distance inhibition of the growth of GFP-tagged L. pneumophila Lens by 1-undecene. A vial containing 1-undecene was placed on the agar in the central well. The opening of the vial is facing left in the picture. The wells on either side were inoculated with GFP-tagged L. pneumophila Lens. Larger or smaller colonies are observed depending on the concentration of 1-undecene used. Top panel: control without 1-undecene; central panel: the vial was filled with 4 mM 1-undecene; bottom panel: the vial was filled with 2 mM 1-undecene. Plates were incubated 72 h at 28°C and bacterial growth was qualitatively visualized. The initial plating of the bacterial suspension on the agar plate is indicated by yellow arrows. Images are representative of more than three independent experiments. Error bars indicate SDs. A.U. indicates arbitrary units.

To further identify the volatile compound(s) responsible for the antagonistic activity against L. pneumophila, random mini-Tn*5* transposon mutagenesis was used to generate mutants in P. fluorescens MFE01. The library consisted of 2,000 randomly picked mutants that were individually screened using the two-petri-dish assay with a GFP-expressing L. pneumophila Lens. This screening led to the identification of five mutants unable to inhibit the growth of L. pneumophila Lens at distance (Fig. 3A). The GFP fluorescence intensity varied according to the mutant tested and for some of them (3G4, 4G2, and 3H2), a weak inhibiting activity was conserved. Nevertheless, the fluorescence intensity in the presence of 3H5 and 3H8 mutants was as high as for the control without MFE01, indicating they have no significant effect on the growth of L. pneumophila. Using a vectorette PCR method, the mini-Tn*5* transposon insertion site was identified for each MFE01 mutant (Table 2). Two mutant pairs collected from different petri dishes harbored the same insertion site, 3H2/3G4 in a putative ribosomal *N*-acetyltransferase-encoding gene and 3H5/3H8 in the *trpE* gene. Both 3H5 and 3H8 have the same Tn*5* insertion site and were more likely clonal. Since 3H5 and 3H8 mutants were the only strains that had completely lost the at-distance inhibiting phenotype, we chose one of them, strain 3H5, to further characterize the differences in the volatiles produced compared to the wild-type MFE01 strain.

**TABLE 2:** Genes interrupted by the Tn*5* transposon and known functions

Mutant	Interrupted gene	Gene product	GO molecular function^*a*^
3H2 and 3G4	NA	Putative ribosomal N-acetyltransferase	N-terminal peptidyl-serine acetylation
4G2	NA	Hypothetical protein	NA
3H5 and 3H8	*trpE*	Anthranilate synthase component 1	Anthranilate synthase activity/tryptophan biosynthetic process

a GO, the Gene Ontology Resource (http://geneontology.org/). NA, not applicable.

We first analyzed the volatilome of MFE01 by SPME/GC-MS. This strain produces 18 volatile organic compounds (VOCs) when grown on LB agar medium at 28°C, including acids, alkanes, alkenes, ketones, and one aldehyde (Table 3). The main VOCs produced by MFE01 were methyl cis-2-butenoate, 4-hydroxybutanoic acid, cycloundecene, 1-undecene, and 2-undecanone. We then determined the molecules directly involved in the antagonistic activity against *Legionella* by comparing the volatilomes of MFE01 and its 3H5 mutant at different times during growth (Fig. 3B to D). The 3H5 strain produces less 1-undecene and, to a lesser extent, less 2-undecanone and 2-tridecanone than the MFE01 strain

**TABLE 3:** Volatile organic compounds identified from the volatilome of P. fluorescens MFE01 by headspace SPME/GC-MS

Retention time (min)	Compound	Peak area (A.U.)^*a*^
5.086	Methyl cis-2-butenoate	2.36 × 10^7^
11.239	1-Nonene	4.70 × 10^6^
11.979	4-Hydroxybutanoic acid	2.46 × 10^7^
14.749	Cyclodecene	3.26 × 10^6^
17.904	Cycloundecene	2.49 × 10^7^
18.061	1-Undecene	2.28 × 10^9^
18.482	5-Undecene	5.69 × 10^6^
18.758	2-Undecene	3.56 × 10^6^
21.098	1-Dodecene	3.19 × 10^6^
21.350	Dodecane	2.99 × 10^6^
21.519	Decanal	1.03 × 10^6^
23.538	Cyclotridecene	8.48 × 10^6^
23.943	Undecanal	2.75 × 10^6^
23.986	2-Undecanone	2.05 × 10^7^
26.807	Tetradecane	2.58 × 10^6^
29.577	2-Tridecanone	7.19 × 10^6^
32.763	Hexadecane	1.23 × 10^6^
35.683	Heptadecane	4.40 × 10^6^

a A.U., arbitrary units.

### Inhibition of the growth of L. pneumophila is mainly due to 1-undecene.

**FIG 4:**
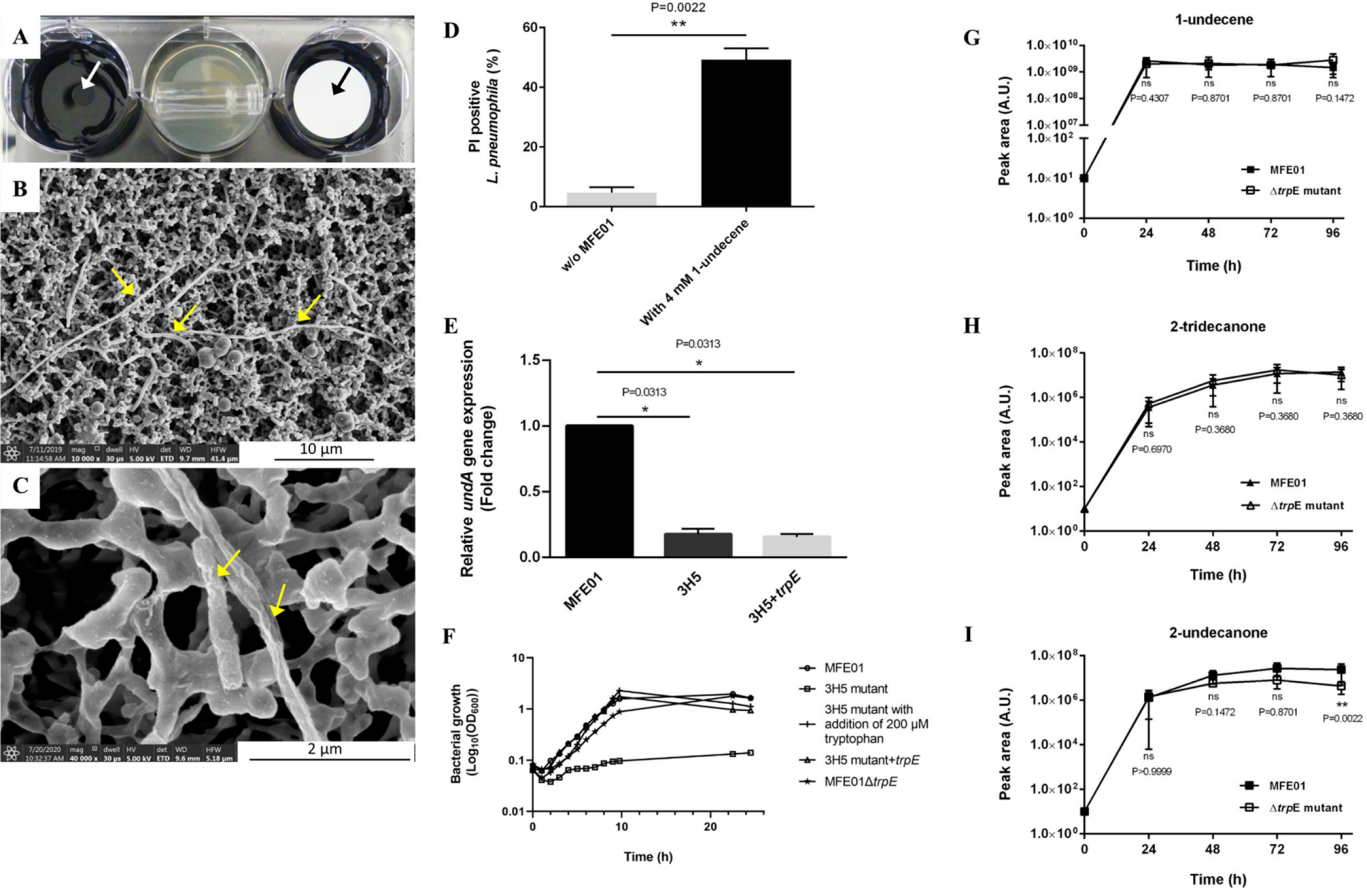
Lytic potency of 1-undecene and production in both 3H5 and Δ*trpE* mutants. (A to C) Scanning electron micrographs of L. pneumophila grown on nitrocellulose filter (0.22 μm) at 28°C for 72 h in the presence of a 1-undecene. The central well contained LB, onto which was placed a vial filled with 4 mM 1-undecene. The opening of the vial is oriented to the right in the picture. The wells on each side were inoculated with GFP-tagged L. pneumophila Lens either on BCYE agar or on nitrocellulose filter. Spots of GFP-tagged L. pneumophila are indicated by black and white arrows. Damaged bacilli are indicated by yellow arrows. Pictures are representative of four independent assays. (D) Envelope permeabilization of GFP-tagged L. pneumophila Lens. Bacteria were harvested from nitrocellulose filter (0.22 μm), labeled 15 min with propidium iodide (PI) and were subjected to flow cytometry analysis. w/o MFE01: test without MFE01. **, *P < *0.01, compared to the permeabilization in the absence of MFE01 (Mann-Whitney U test). Data represent the mean (± SD) of three independent experiments, each performed in duplicate. (E) Relative expression of *undA* in MFE01 and mutants assayed by RT-qPCR. *, *P < *0.05, compared to MFE01 (one-sample Wilcoxon test). Data represent the mean (± SD) of three independent experiments, each performed in duplicate. (F) Determination of tryptophan auxotrophy in P. fluorescens MFE01, 3H5 and its complemented strain. Bacteria were grown in 25 ml MOPS minimal medium with no tryptophan (initial OD_600_ 0.08). To assay the tryptophan-dependent growth of 3H5, tryptophan was added to a final concentration of 200 mM at 3 h after inoculation. The growth curves were repeated three times and one representative curve for each strain is presented here. (G to I) SPME/GC-MS kinetics of 1-undecene (G), 2-undecanone (I), and 2-tridecanone (H) production by the P. fluorescens MFE01 Δ*trp*E mutant. Each strain was cultured in LB medium for 24 h and a suspension diluted at OD_600_ = 1 was deposited on a sterile GC vial filled with LB agar. Vials were then incubated at 28°C and analyzed at different time points by SPME/GC-MS. **, *P < *0.01, compared to the peak area obtained with MFE01 (Mann-Whitney U test). A *P* value > 0.05 was considered to be statistically nonsignificant (ns). Data represent the mean (± SD) of three independent experiments, each performed in duplicate. Error bars indicate SDs. A.U. indicates arbitrary units.

Using chemical standards, we determined that each compound is individually able to inhibit at distance the growth of L. pneumophila in a molar range of concentration, but only 1-undecene was found to exert a long-distance inhibition at lower concentrations (between 2 and 4 mM) (Fig. 3E). This concentration of 1-undecene seems biologically relevant because it is of the same order of magnitude as that produced during MFE01 growth in GC vials (i.e., 220 μM, Fig. S1 in the supplemental material, Table 3). The remaining difference could be attributed to the use of the standard, which does not mimic the accumulation of VOC *in vitro* and makes the test less sensitive. We visualized by SEM the morphology of L. pneumophila after exposure with 4 mM 1-undecene for 72 h (Fig. 4A to C). Only a few filamentous bacilli could be observed on the filter (Fig. 4B). At a higher magnification, the morphology of the bacilli was also altered and the presence of invaginations could be noted (Fig. 4C). Nevertheless, bacteria remained identifiable and appeared less damaged than in the presence of MFE01. A PI staining of L. pneumophila showed that just over 50% of the population recovered from the filter had a damaged envelope (Fig. 4D). These data are in agreement with those obtained with MFE01, as a small amount of 1-undecene induces similar effects on L. pneumophila as an exposure to the wild-type bacterium (Fig. 2).

The *undA* gene, reported as responsible for 1-undecene production in Pseudomonas, was discovered a few years ago (35). Therefore, we quantified the expression of the homologous gene in MFE01 by quantitative reverse transcriptase PCR (RT-qPCR). The *undA* transcripts were downregulated by 5.6-fold in a 3H5 mutant after 24 h of incubation at 28°C compared to the wild-type strain, supporting our previous results concerning a decrease in 1-undecene production (Fig. 4E). Overall, our results demonstrate that 1-undecene is the main volatile compound inhibiting the growth of L. pneumophila Lens, and its production/secretion is strongly affected in the 3H5 transposition mutant.

### TrpE is not directly involved in the anti-*Legionella* activity of MFE01.

In Pseudomonas species, the enzyme TrpE is known to transform chorismic acid into anthranilic acid, a precursor of tryptophan (36–38). To confirm its direct implication in the anti-*Legionella* activity of MFE01, *trpE* was expressed in *trans* in the 3H5 mutant. Successful transformation using the pPSV35-*trpE* vector was proved by the complementation of the tryptophan auxotroph phenotype observed in the 3H5 mutant (Fig. 4F). However, the wild-type volatile anti-*Legionella* interference phenotype was not restored in the 3H5+*trpE* strain (Fig. S2). In addition, in the 3H5+*trpE* strain, the *undA* transcripts were downregulated similarly to those of the 3H5 mutant (Fig. 4E).

In the same vein, we constructed an MFE01Δ*trpE* in-frame deletion mutant by directed mutagenesis and reintroduced in this mutant the *trpE* gene in *trans* to obtain the MFE01Δ*trpE*+*trpE* strain. Surprisingly, this *trpE* deletion mutant was not a tryptophan auxotroph and was still able to inhibit the growth of *L pneumophila* at distance like the wild-type strain (Fig. 4F, Fig. S2). In addition, no differences were recorded between the volatilomes of MFE01Δ*trpE* and MFE01 strains, even for the three compounds of interest: 1-undecene, 2-undecanone, and 2-tridecanone (Fig. 4F to I). Given that neither complementation nor creation of a *trpE* deletion strain altered the volatile inhibition, TrpE is not directly involved in the capacity of MFE01 to inhibit the growth of L. pneumophila Lens at distance.

**FIG 5:**
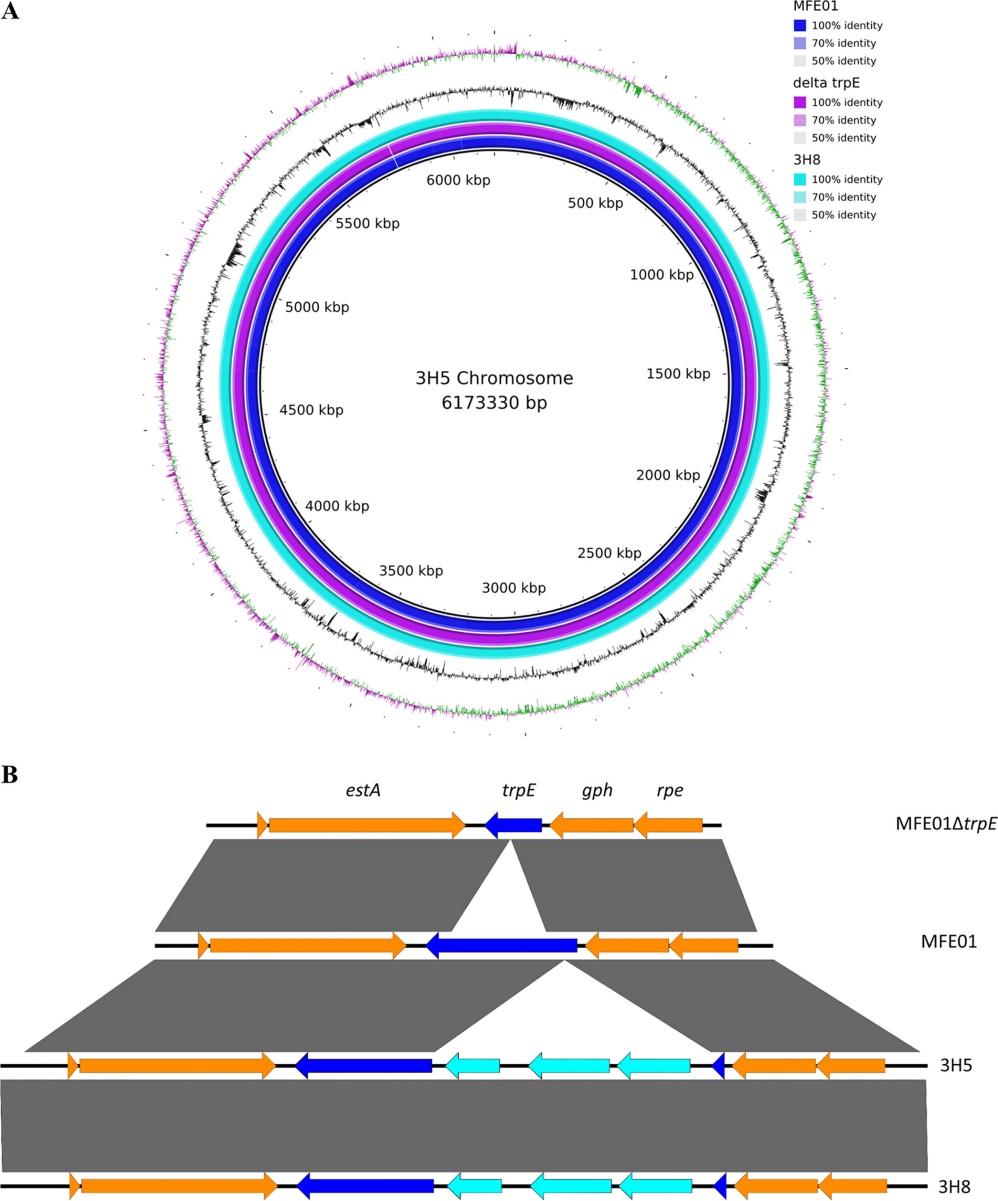
Genome analysis of P. fluorescens MFE01 and its mutants. (A) BRIG representation of BLAST comparisons from MFE01 wild-type, MFE01Δ*trp*E, and 3H8 genomes against the assembled genome of strain 3H5. (B) Easyfig image showing linear view of the *trpE* region with and without the presence of the mini-Tn*5*. Orange arrows represent coding regions surrounding *trpE*, dark blue arrows represent the *trpE* coding region, and light blue arrows represent the Tn*5* coding regions. GenBank accession number for *trpE*: MT542516.

**FIG 6:**
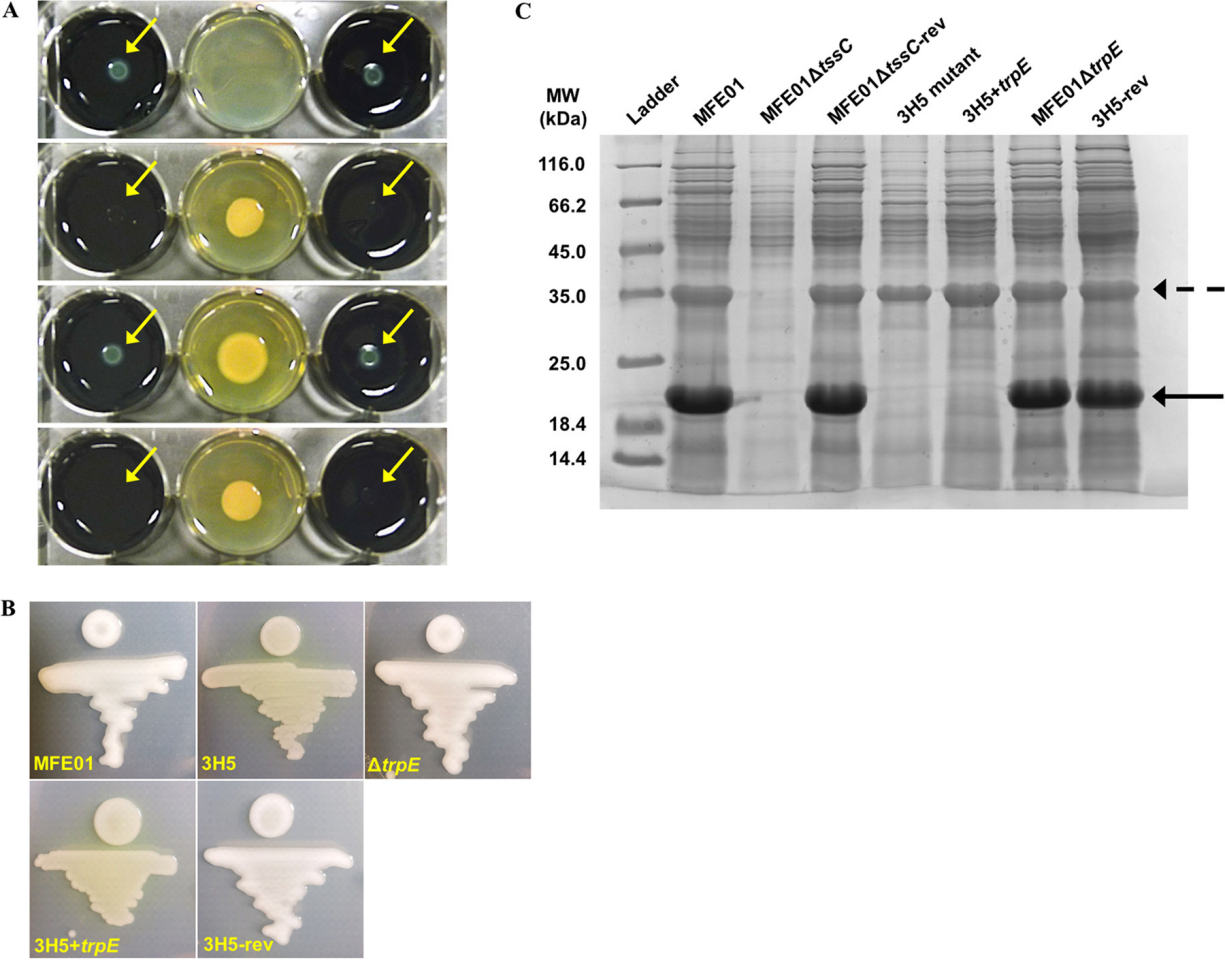
The 3H5 reversion mutant (3H5-rev) shows restoration of all observed phenotypes. (A) Volatile interference between P. fluorescens MFE01, 3H5, or 3H5-rev and GFP-tagged L. pneumophila Lens using the 6-well plate assay. First panel on top: control without MFE01. Second panel: presence of MFE01. Third panel: presence of the 3H5 mutant. Last panel at the bottom: presence of the 3H5-rev strain. The initial plating of the bacterial suspension on the agar plate is indicated by yellow arrows. Images are representative of more than three independent experiments. (B) Mucoid phenotype of MFE01 and its mutants grown on LB agar at 28°C. The images shown are representative of three assays. (C) Supernatants of cultures in stationary growth phase, grown at 28°C, were concentrated by trichloroacetic acid (TCA) and proteins were analyzed by SDS-PAGE (15% separating gel) and Coomassie staining. Flagellin, with an approximate molecular mass of 35 kDa, is indicated by a dotted arrow, while Hcp proteins, with an approximate molecular mass of 21 kDa, are indicated by a solid arrow.

Such results therefore leave a gap in our understanding of the 3H5 mutant and what causes the difference between the volatiles produced. We therefore sequenced the genomes of all strains to look for mutations that could explain the phenotypes obtained. Genomes of MFE01, 3H5, 3H8, and MFE01Δ*trpE* were sequenced using both short-read and long-read sequencing. Finished genomes were obtained by hybrid assembly of these short and long reads (Fig. 5A). This whole genome sequencing confirmed in both 3H5 and 3H8 mutant strains the insertion of only one copy of the mini-Tn*5*, occurring in a gene assigned to *trpE* (Fig. 5B) (GenBank accession number MT542516). Secondly, short-read mapping did not reveal any single nucleotide polymorphism (SNP) between the wild-type MFE01 and its three derivative strains. Except for the *trpE* deletion or the Tn*5* insertion, comparison of hybrid assemblies did not reveal any genomic rearrangement for the four strains (Fig. 5A). Thus, we managed to obtain a reversion mutant by chromosomal reintroduction of *trpE* to its original location, which implies removal of the transposon and restoration of the native *trpE* locus. This mutant (named 3H5-rev) showed a restoration of the capacity to inhibit the growth of L. pneumophila at distance and the reversion of tryptophan auxotrophy (Fig. 6A, Fig. S3). Our data thus demonstrate that the decrease in VOC production and, in particular 1-undecene, which leads to the loss of anti-*Legionella* activity, is due to polar effects resulting from the insertion of mini-Tn*5* into *trpE*.

### Mucoid phenotype assigned to the 3H5 mutant is associated with a defect in the type 6 secretion system.

The only visible phenotype observed in the 3H5 mutant is the loss of the mucoid phenotype when grown on LB agar at 28°C and compared to the wild-type strain (Fig. 6B). In the 3H5+*trpE* strain, the mucoidy was not restored, whereas the MFE01Δ*trpE* strain also exhibited a mucoid phenotype (Fig. 6B). However, the 3H5-rev strain shows a restoration of the mucoid phenotype. The loss of mucoidy may therefore correlate with the loss of anti-*Legionella* activity and vice versa.

In MFE01, a loss of mucoidy was previously linked to a defect in the type 6 secretion system (T6SS) (39–41). Thus, we investigated if the T6SS was also impaired in 3H5 and if a MFE01-T6SS mutant (MFE01Δ*tss*C) still had the capacity to inhibit the growth of *Legionella* at distance. Hcp (hemolysin coregulated protein) proteins, which are constituents of the T6SS inner tube (40), are normally released in the MFE01 culture supernatant. As expected, MFE01Δ*tssC* did not secrete the Hcp proteins (band at 21 kDa) in the medium, unlike strains MFE01 and MFE01Δ*tssC*-revertant (Fig. 6C). No bands were detected at 21 kDa in the analysis of the 3H5 and 3H5+*trpE* strains, indicating that 3H5 is impaired in the secretion of Hcp and that the *trpE* expression in *trans* did not restore this secretion. In the same vein, the deletion mutant MFE01Δ*trpE* still secreted large amounts of Hcp, indicating that the *trpE* gene expression is not involved in the T6SS regulation (Fig. 6C). However, the 3H5-rev strain secretes Hcp proteins in the supernatant, indicating that the defect observed in the T6SS is due to the polar effects resulting from the insertion of the transposon in *trpE*.

Finally, we observed that both MFE01Δ*tssC* and MFE01Δ*tssC*-revertant were able to aerially inhibit the growth of L. pneumophila Lens at distance (Fig. S4). Taken together, these data indicated that the loss of mucoidy is associated with a defect in the secretion of Hcp proteins and that these phenotypes are driven by the polar effect of the transposon insertion in *trpE* and are not linked to the anti-*Legionella* activity.

## DISCUSSION

Although L. pneumophila is one of the most closely monitored waterborne pathogenic bacteria, the range of natural active compounds studied to bypass chemical agents such as chlorine remains limited (42). Recent studies have shown the importance of the genus Pseudomonas as a source of anti-*Legionella* compounds without ever mentioning VOCs (14, 15). To date, only biosurfactants have been described as anti-*Legionella* compounds. In this study, we demonstrated for the first time the unexpected activity of a Pseudomonas fluorescens strain named MFE01 that inhibits the growth of Legionella pneumophila at distance. Therefore, the volatilome of MFE01 was analyzed and a total of 18 VOCs were identified. They were grouped into 9 chemical families commonly reported in the literature, including alcohols, aldehydes, alkenes, alkanes, alkynes, esters, ketones, terpenoids, and sulfur-containing compounds (43, 44). Our analysis also revealed that 1-undecene was the major VOC produced by MFE01. This compound has already been detected in the volatilomes of several bacteria and, in particular, in those of many Pseudomonas species (43, 45–49). Together with some others, such as 2-butanone, 1-undecene was notably proposed to serve as a biomarker for clinical investigation of the presence of P. aeruginosa during respiratory infections (43, 50, 51). In addition, 1-undecene has also been previously reported to promote plant growth (45), to exert antifungal activity (52), and to negatively affect growth and survival of nematodes (53). Nevertheless, this compound has never been studied for its antibacterial activity. Our data revealed that the volatilome of MFE01, through the dispersion of 1-undecene, 2-undecanone, and 2-tridecanone, in particular, displays a narrow antibacterial spectrum directed toward clinical and environmental *Legionella* species, including Legionella pneumophila, the etiological agent responsible for severe forms of lung infections known as legionellosis. To go further, we demonstrated the bactericidal activity of the molecules comprising the MFE01 volatilome on L. pneumophila and, in particular, of 1-undecene, which leads to the lysis of bacteria. This is the first time that long-distance bacterial lysis by VOCs has been proved. Some papers have reported antibacterial effects of microbial VOCs (29, 54–56) but, to date, few have observed a direct bactericidal effect (44). This sensitivity to the VOCs produced by MFE01 is widespread in the *Legionella*, although the reason why some strains, including *L. feelei* or L. pneumophila strain Chicago 8, are less sensitive or completely tolerant remains unclear. Nevertheless, these data led us to hypothesize that many *Legionella* species, unlike the other bacteria tested, will possess some specificities in the envelope that makes them particularly sensitive to the action of these compounds. A detailed overview of the main characteristics of the *Legionella* cell envelope was presented a few years ago (57) and some have been suspected to be responsible for the high sensitivity of this bacterium to antimicrobial peptides and detergents (42). These specificities include a unique structure of the lipopolysaccharide with large proportion of *O*- and *N*-acetyl groups, a high level of phosphatidylcholine, and branched-chain fatty acids. However, the way in which these characteristics could modulate the activity of active molecules has not yet been elucidated (58, 59). VOCs could induce ultrastructural changes in target cells, such as alterations in the cell wall and membranes, that could lead to changes in cell permeability (32, 44). Since the growth of all non-*Legionella* spp. bacteria tested in this study was not affected by the VOCs of MFE01, this supports the hypothesis of a key role played by components of the *Legionella* cell envelope.

A Tn*5*-transposon MFE01 mutant collection was constructed and five mutants unable to inhibit at distance the growth of L. pneumophila Lens were selected. We focused our attention on the 3H5 mutant in particular because of the total loss of the long-distance inhibitory phenotype associated with a decrease in 1-undecene production. The Tn*5*-transposon insertion disrupted a gene assigned to *trpE*, whose product encodes an enzyme responsible for the production of anthranilate, a precursor in the biosynthesis pathway of tryptophan (36). The 3H5 mutant exhibited a tryptophan auxotroph phenotype, which is in agreement with previous findings describing this phenotype after a deletion of the *trpE* gene in P. aeruginosa (37, 38). The homologous gene to *undA* was found to be significantly downregulated in the 3H5 mutant compared to the wild-type strain. This gene is well conserved in several species of Pseudomonas and was described to be responsible for 1-undecene biosynthesis, converting medium-chain fatty acids (C10 to C14) into their corresponding terminal olefins using an oxygen-activating, nonheme iron-dependent mechanism (35). Those pieces of data are in agreement with our findings about the diminution of 1-undecene in 3H5 and reinforce the key role played by this VOC as an anti-*Legionella* compound. Although it was possible to successfully complement the tryptophan auxotroph phenotype using a *trpE*-bearing plasmid vector, the wild-type volatile anti-*Legionella* interference phenotype was not restored in the 3H5 mutant. Intriguingly, an MFE01Δ*trpE* in-frame deletion mutant constructed by site-directed mutagenesis and verified by whole-genome sequencing was still able to inhibit the growth of L. pneumophila at distance, similarly to the wild-type strain, although it is prototrophic for tryptophan. Indeed, this *trpE* deletion did not affect the number of VOCs emitted by the strain and had only a small effect on their released amounts. Given that a *trpE* in-frame deletion did not alter the volatile inhibition, we conclude that TrpE is not directly related to the anti-*Legionella* phenotype. The 3H5 strain is defective in tryptophan biosynthesis and its phenotype can be complemented by the *trpE* gene expression in *trans*. This seems in contrast with the tryptophan autotrophy of the MFE01Δ*trpE* mutant. This would suggest that *trpE* is both essential and not essential for tryptophan production. Several hypotheses might explain this apparent contradiction. First, in the MFE01 genome we found another *trpE*-like gene, the expression of which may complement the in-frame *trpE* mutation of MFE01Δ*trpE* mutant but not the disruption of *trpE* in 3H5, due to polar effects of the Tn*5* insertion. Second, a study has already documented the reversion of tryptophan auxotrophy in an in-frame *trpE* mutant in P. aeruginosa (37). The phenomenon was due a G-to-A change at nucleotide 1041 of the *pqsC* gene; however, this gene is absent in MFE01. Moreover, comparison of the whole-genome sequences of 3H5 and MFE01Δ*trpE* did not provide an obvious explanation for the tryptophan phenotype discrepancy between the two strains. Finally, despite a deletion spanning two-thirds of the gene, MFE01Δ*trpE* might express a truncated protein that retained sufficient structure to carry an efficient role based on the use of chorismate as a substrate.

At this point, to fill the gap in our understanding of mutant 3H5 and what is causing the difference in the volatiles produced, we managed to study polar effects. Indeed, it is well known that a transposon insertion, in addition to disrupting the function of a gene, can also have polar effects on the expression of adjacent genes or operons, due to the presence of a common or intermittent promoter or by interfering with transcription or translation of mRNA (60). A reversion mutant was obtained by chromosomal reintroduction of *trpE* at the transposon site. This strain (3H5-rev) showed a total restoration of the anti-*Legionella* activity together with the mucoidy and tryptophan autotrophy, confirming that all phenotypes resulted from polar effects induced by the Tn*5* transposon insertion into *trpE*. Interestingly, a link between mucoidy and type six secretion system (T6SS) was previously established in MFE01 without being fully elucidated (45, 47). To further investigate this mucoid phenotype, we tested whether T6SS could be involved in anti-*Legionella* activity. The Tn*5* insertion led to an alteration in Hcp proteins secretion and loss of the T6SS-associated mucoid phenotype, as previously described for the T6SS mutant MFE01*Δtss*C (45–47). However, the MFE01Δ*tssC* strain was still able to aerially inhibit the growth of L. pneumophila Lens at distance and produce VOCs, indicating there is no direct link between the bacterial inhibition and T6SS.

This work brings new insights into the growing field of microbial volatiles. A long-distance bacterial lysis is a spectacular mechanism that highlights the increased sensitivity of *Legionella* sp. to membrane-active compounds. Our data should kickstart the identification of the molecular target(s) that make this sensitivity possible in order to develop innovative compounds or solutions to fight this opportunistic pathogen.

## MATERIALS AND METHODS

### Bacterial strains, plasmids, and culture conditions.

Bacterial strains used in this study are listed in Table S1 in the supplemental material. P. fluorescens strain MFE01 was cultured at 28°C for 24 h either on lysogeny broth (LB) with 5 g/liter NaCl or MOPS (morpholinepropanesulfonic acid) agar plates or in liquid medium under shaking (180 rpm). When needed, 25 to 50 μg/ml gentamicin and 1 mM IPTG (isopropyl-β-D-thiogalactopyranoside) were added to media. The MOPS minimal medium is a MOPS-buffered salts base (50 mM MOPS [pH 7.2], 93 mM NH_4_Cl, 43 mM NaCl, 3.7 mM KH_2_PO_4_, 1 mM MgSO_4_, 3.5 mM FeSO_4_·7H_2_O) supplemented with 20 mM succinate as the sole source of carbon. The MOPS buffer is a MOPS medium without carbon source.

*Legionella* strains were cultured at 37°C for 72 h either on buffered charcoal yeast extract (BCYE) agar plates or in buffered yeast extract (BYE) liquid medium under shaking (180 rpm). Other strains were grown at 37°C for 24 h either on LB agar plates or in broth. Plasmids used in this study are listed in Table S1. All chemicals were purchased from Sigma-Aldrich (St. Louis, MO, USA) unless otherwise stated.

### Anti-*Legionella* activity tests.

In a first step, the spot-on-lawn assay was performed as described previously (15). A 10-μl drop of a P. fluorescens MFE01 preculture (adjusted to a concentration of 10^9^ CFU/ml with LB) was spotted onto the center of the agar plate prior to incubation at 28°C for 72 h. In a second step, a two-petri-dish assay adapted from a previous study was used (20). An uncovered 3.5-cm Petri dish was placed in the center of a 9-cm classical Petri dish. The resulting external ring was filled with 20 ml BCYE while the inner small Petri dish was filled with 4 ml LB agar. A P. fluorescens MFE01 culture (diluted to a final optical density at 600 nm [OD_600_] of 0.1) or LB medium (control) was deposited in the central petri dish as a source of volatile compounds. Then, two 20-μl drops of a 48-h-old culture of L. pneumophila (adjusted to a final OD_600_ of 1) were spotted onto the external agar ring. The small petri dish was sealed with a lid or not, depending on the tested condition. The large petri dish was finally closed and incubated for 72 h at 28°C. Volatile activity was qualitatively estimated as a function of the growth of L. pneumophila (presence or absence of colonies). A previously designed 6-well plate quantitative long-range inhibition assay was also used in this study (14). Plates were incubated at 28°C for 72 h to 96 h. Anti-*Legionella* activity was quantified by measuring the fluorescence of growing GFP-tagged L. pneumophila Lens (61) (TriStar2 LB 942 Microplate Reader, Berthold Technologies, BadWildbad, Germany). For analysis of the bacterial envelope integrity, a flow cytometry-based assay was carried out according to a method detailed elsewhere (62). A total of 10,000 events were recorded per condition.

### Analyses of volatile organic compounds by GC-MS.

Sterile vials (20 ml) adapted to GC-MS were prepared by adding 4 ml of LB agar horizontally and 6 ml vertically. A 40-μl drop of a 24-h-old culture of P. fluorescens MFE01 or mutants (adjusted to a final OD_600_ of 1) was spotted on the agar; vials were then sealed and incubated at 28°C for 0, 24, 48, 72, or 96 h before being analyzed by GC-MS. The injection vials were sealed with an aluminum crimp cap provided with a polytetrafluoroethylene/silicone septum (Agilent Technologies, Massy, France) and left in the incubator until analysis. Prior to analysis, the 100 μm SPME polydimethylsiloxane (PDMS) fiber (Sigma-Aldrich, St. Louis, MO, USA) was preconditioned in the bake-out oven of the Gerstel injector as recommended by the manufacturer (250°C for 30 min). Extraction duration profiles were carried out for 5 min at 28°C. Desorption time was set at 5 min in the GC injection port sets at 250°C. GC-MS analyses were performed in a 7890B Agilent gas chromatograph. Helium was used as the carrier gas at a constant flow rate of 1.2 ml/min. The injector operated in the splitless mode (50:1) and its temperature was set at 250°C. The separation of volatile compounds was performed on a Zebron ZB-5HT Inferno column (30 m × 0.25 mm, 0.25 μm; Phenomenex, USA). The oven temperature program started at 40°C (held for 5 min), was raised at a rate of 5°C/min to 150°C (held for 2 min), and then raised at a rate of 4°C/min to 230°C (held for 2 min). The detection was performed by an Agilent 7000C triple quadrupole mass sets in positive electron impact mode (+EI) with 70 eV of electron energy. The electron multiplier was set by the auto-tune procedure. MS data were collected in a full scan mode over the *m/z* range from 35 to 400. Transfer line temperature was set at 300°C, the first and second quadrupole (Q1, Q2) temperature at 150°C, and the ion source temperature at 230°C. All samples were analyzed twice with independent biological triplicates.

### Generation of P. fluorescens MFE01 transposon mutants.

The donor strain, Escherichia coli S17-1 (63), contained a suicide plasmid pAG408 (ATCC 87653; 5.7 kb) (64) with a mini-Tn*5* transposon. This plasmid was transferred into P. fluorescens MFE01 recipient cells by biparental mating. Bacterial cells were mixed and spotted onto sterile nitrocellulose filters, which were placed on LB agar plates and incubated overnight at 37°C. The mating mixture was suspended in 1 ml of sterile physiological water and 0.1-ml aliquots were spread on LB agar plates supplemented with gentamicin (50 μg/ml), to select for the presence of the integrated transposon, and rifampin (25 μg/ml) to kill the E. coli S17-1 donor bacteria and to ensure the selection of MFE01.

### Determination of transposon insertion sites by vectorette PCR.

Genomic DNA of mutants was extracted using the NucleoSpin tissue kit (Macherey-Nagel) as recommended by the manufacturer. Samples were then treated with 10 μg/ml RNase A at 37°C for 30 min before being digested for 2 h with the StuI restriction enzyme (Promega, Charbonnières-les-Bains, France). Newly generated DNA fragments were ligated to a DNA vectorette using the following mix (total of 20 μl): 1.5 μl of 50 ng/μl genomic DNA, 1 μl 10× buffer, 1 μl vectorette, 0.1 μl T4 DNA ligase (3u/μl), and 16.4 μl of water. The ligation reaction was performed overnight at 15°C. The DNA vectorette was built from an equimolar mixture of AV21 and AV22 primers (2 μM each) (Table S2). The DNA mixture was then incubated at 65°C for 5 min before addition of 1 mM MgCl_2_ in order to stabilize the double-stranded DNA formed. The vectorette is only partially double stranded and contains a central mismatched region so that it can be amplified only if it is attached to the DNA sequence of interest (65). To detect the Tn*5* transposon, DNA samples were PCR amplified with primers AV24/Iseq or AV24/Oseq and 5% dimethyl sulfoxide (DMSO) in the final mix. Amplification was carried out as follows after a first step of denaturation at 95°C for 2 min: denaturation at 95°C for 1 min, annealing at 58°C for 1 min and extension at 72°C for 3 min, for a total of 30 cycles, followed by a final elongation at 72°C for 10 min. Then, PCR products were subjected to 1% agarose gel electrophoresis and further purified using a NucleoSpin Gel and PCR clean-up kit (Macherey-Nagel, Bethlehem, PA, USA). DNA sequencing was completed with the ABI Prism BigDye Terminator v3.1 sequencing kit (Applied Biosystems, Carlsbad, CA, USA) and then analyzed by an automated DNA sequencer (ABI Prism 3130 Genetic Analyzer, AppliedBiosystems, Carlsbad, CA, USA).

### Phenotypic characterization of transposition mutants.

After an overnight culture, the P. fluorescens MFE01 concentration was adjusted to an OD_600_ of 1. Mucoid phenotype was then evaluated by spreading bacteria on 1.5% agar LB plates and incubating them at 28°C for 24 h.

### Construction of a 3H5+*trpE* strain.

The *trpE* gene was amplified from the P. fluorescens MFE01 genome using Phusion High-Fidelity DNA polymerase (New England BioLabs, Ipswich, MA, USA) with the AnthrSyn-SacI-F and AnthrSyn-XbaI-R primers (Table S2). The PCR conditions were as follows: an annealing temperature of 60°C, an extension time of 1 m 35 s and 35 cycles. The amplified fragment (<1,500 bp) and the pPSV35 shuttle vector (66) were digested by SacI and XbaI (New England BioLabs) at 37°C for 1.5 h and then inactivated at 65°C. The *trpE* gene was inserted into the pPSV35 by ligation at 25°C overnight (T4 DNA Ligase, New England BioLabs), and the resulting pPSV35-*trpE* was used to transform E. coli Top10 cells by thermal shock. Plasmid DNA was isolated using the QIAprep Spin miniprep kit (Qiagen, Courtaboeuf, France) and was verified by DNA sequencing using the Seq-pPSV35-F and Seq-pPSV35-R primers (Table S2). An overnight culture of 3H5 was washed twice with 1 ml cold sterile water before transformation with 150 ng of pPSV35-*trpE* by electroporation in 2-mm electroporation cells at 1.8 KV for 5 ms (Bio-Rad Gene Electroporator). After addition of LB medium, mixtures were incubated for 1.5 h at 28°C with shaking (180 rpm). Transformed strains 3H5+*trpE* were selected by plating bacteria on both LB agar plates supplemented with 50 μg/ml gentamicin and 1 mM IPTG and on MOPS agar plates supplemented with 200 mM tryptophan. Transformed strains were verified by PCR using the Seq-pPSV35-F and Seq-pPSV35-R primers (Table S2).

### Disruption of the *trpE* gene in P. fluorescens MFE01.

A markerless *trpE* mutant was constructed by overlap PCR mutagenesis, as described elsewhere, with modifications (39). This in-frame *trpE* deletion was achieved by PCR with the Muta1-AnthrSyn-F and Muta2-AnthrSyn-XbaI-R primers (>700 bp product, amplicon A) and the Muta3-AnthrSyn-XbaI-F and Muta4-AnthrSyn-R primers (>750 bp product, amplicon B) (Table S2). The polymerase used was the Phusion High-Fidelity DNA polymerase (New England BioLabs). The PCR products (amplicons A and B) obtained correspond to the upstream and downstream parts, respectively, of the *trpE* gene of MFE01, each carrying an XbaI restriction enzyme site sequence at the end. PCR parameters were as follows: annealing temperature, 55°C; extension time, 1 min; 30 cycles. Then, both amplicons A and B were digested by XbaI (New England BioLabs; 37°C, 1.5 h and inactivated at 65°C) and ligated overnight at 25°C (T4 DNA Ligase, New England BioLabs [NEB]). A third PCR was performed using purified ligature product (GeneJet PCR purification kit, Thermo Fisher Scientific) allowing the *trpE* deletion (924 pb) after PCR with the Muta1-AnthrSyn-F and Muta4-AnthrSyn-R primers. The PCR parameters were as follows: annealing temperature, 55°C; extension time, 2 min; and 35 cycles. This PCR fragment was introduced into the conjugative suicide vector pAKE604 (67) (Table S1). The resulting plasmid, pAKE604Δ*trpE*, was verified by sequencing and was then transferred into MFE01 by biparental mating as described above. The mutant containing the *trpE* deletion was verified by DNA sequencing (using the Muta1-AnthrSyn-F and Muta4-AnthrSyn-R primers) and named MFE01Δ*trpE.* Complementation of this mutant using the pPSV35-*trpE* vector was performed exactly as described for the construction of the 3H5+*trpE* strain.

### Construction of the revertant strain 3H5-rev.

Each PCR was performed under standard conditions using Phusion High-Fidelity DNA polymerase (NEB). The *trpE* amplicon (wild-type gene and environment) was amplified from P. fluorescens strain MFE01 with Muta1-AnthrSyn-F and Muta4-AnthrSyn-R primers (Table S2) using the following PCR parameters: 61°C for 15 s, 72°C for 2 min; 30 cycles. The resulting *trpE* amplicon was introduced into the pAKE604 suicide vector, previously digested by SmaI (blunt-ended), and ligated with T4 DNA ligase. The construct was verified by sequencing and then introduced into E. coli S17-1 (63). The recombinant plasmid was transferred by biparental mating: recipient 3H5 and the S17-1 strain containing pAKE604-*trpE* were mixed at the same ratio and spotted onto LB agar medium and incubated at 37°C overnight. The biomass mixture was resuspended in 1 ml sterile physiological saline and 0.1 ml of the cell suspension spread on LB agar plates supplemented with rifampin (50 μg/ml, for 3H5 selection and E. coli S17-1 killing) and kanamycin (200 μg/ml, for the selection of cells containing the recombinant plasmid) and incubated at 28°C for 48 h. Colonies were isolated on LB agar plates supplemented with 10% sucrose to select the second homologous recombinants. Colonies were tested for gentamycine (50 μg/ml) and kanamycin sensitivity, corresponding to transposon excision. The resulting revertant strain was verified by DNA sequencing and named 3H5-rev.

### Whole-genome sequencing and analyses.

P. fluorescens genomes were sequenced using both short-read and long-read sequencing. Short-read sequencing was performed using the DNAprep library preparation kit (Illumina, San Diego, USA), and paired-end 2 × 150 bp on a Nextseq 550 system (Illumina). Long-read sequences were also sequenced using Nanopore technology (Nanoporetech, Oxford, UK). Library was prepared using the rapid barcoding kit and sequenced on a Minion R9.4.1 flowcell. Finished genomes were obtained by short read/long read hybrid assembly using Unicycler v0.4.6 software (68). Short read mapping and variant calling were performed using snippy v4.0 software and the finished genome of the strain MFE01 as a reference. Hybrid assemblies were aligned using Mauve software (69). Circular representations of blast similarities between genomes were drawn using BLAST Ring Image Generator (BRIG) v0.95 software (70).

### *In vitro* activity of 1-undecene.

Activity of 1-undecene was measured using the 6-well plate quantitative long-range inhibition assay with UV-sterilized LC vials (Waters, Guyancourt, France). A 2-ml vial was placed on LB agar and filled with various volumes of 1-undecene. The MIC of 1-undecene was defined as the minimal volume required to inhibit the entire growth of GFP-tagged L. pneumophila Lens after incubation at 28°C for 72 h.

### Scanning electron microscopy.

For scanning electron microscopy (SEM) analyses, GFP-tagged L. pneumophila Lens (OD_600_ adjusted to 0.1) was grown in a 6-well plate on a nitrocellulose filter (0.22 μm) (Merck Millipore, Molsheim, France) deposited on BCYE agar in the presence of MFE01, as described above. For cell fixation, filters were removed and immersed in 2.5% glutaraldehyde in 1 M phosphate buffer (pH 7.1) for 1 h. Sample preparation was carried out as previously described (62). After HMDS (hexamethyldisilazane) treatment, the surface of the filter was sputter coated in a vacuum with an electrically conductive 25-nm-thick layer of platinum alloy coating system (LEICA EM ACE600 sputter coater). Images were then recorded with a scanning electron microscope (TENEO VolumeScope, FEI) at 5 kV.

### RNA extraction and RT-qPCR.

Spots from MFE01, 3H5, and 3H5+*trpE* were collected from 6-well plates after 24 h of incubation in the presence of GFP-tagged L. pneumophila Lens. Bacteria were resuspended in 400 μl of buffer (12.5 mM Tris, 5 mM EDTA, and 10% glucose) and 400 μl of saturated phenol (pH 4.6) and 0.4 g of glass beads (0.2 to 0.3 mm diameter; Sigma) were added before cell disruption using a Fastprep instrument (5.5 m/s, 30 s, twice; Thermo Fisher Scientific, Illkirch, France). After centrifugation at 14,000 × *g* for 5 min, 1 ml TRIzol reagent (Thermo Fisher Scientific) was added to supernatants. Samples were incubated at room temperature for 5 min. Total RNA was extracted twice with chloroform. RNA was recovered by precipitation with isopropanol and the pellet washed with 75% ethanol. RNA was then dissolved in nuclease-free water and contaminating DNA was removed using RNase-free DNase I (Turbo DNA-free kit, Thermo Fisher Scientific), according to the manufacturer’s instructions. RNA concentration and purity were measured using the NanoDrop 2000 spectrophotometer (NanoDrop Technologies, USA). RNA quality was verified on the Shimadzu MultiNA capillary electrophoresis system (Shimadzu, Marne-la-Vallée, France), according to the manufacturer’s instructions. The samples were stored at −80°C until analysis. Total RNA was reverse transcribed using the GoScript Reverse Transcriptase kit (Promega, Charbonnières-les-Bains, France) according to the manufacturer’s recommendation. The first-strand cDNA products were used to carry out RT-qPCR. All primer sequences are compiled in Table S2. RT-qPCR was carried out using the LightCycler FastStart DNA Master plus SYBR green I in a LightCycler 480 (Roche Molecular Systems, Pleasanton, CA, USA). Reactions were prepared in a total volume of 10 μl containing 5 μl of 2× SYBR mix, 2 μl of H_2_O, 2 μl of diluted cDNA template, and 0.5 μl of 10 μM primers. PCR cycling was performed at 95°C for 10 min, followed by 45 cycles at 95°C for 10 s, 60°C for 10 s, and 72°C for 10 s. An additional 65 to 95°C step (0.5°C/s) was added to confirm that only a single product was amplified in each reaction. A standard curve was obtained with serial dilutions of DNA extracted from strain MFE01 to calculate the amplification efficiencies for each gene. The Pfaffl method was used to evaluate the relative quantitative variation between strains (71). Expression levels of *undA* were quantified in relation to the *recA* control gene. Gene expression from the wild-type MFE01 strain incubated at 28°C was used as reference. RT-qPCR was performed twice with independent biological triplicates to ensure the accuracy of the results.

### Hcp secretion analysis.

Overnight Pseudomonas cultures (50 ml) were centrifuged at 8,000 × *g* for 40 min at 4°C and the collected supernatants were filtered through a 0.22-μm pore size sterile membrane. Trichloroacetic acid was added to the filtered supernatants to a final concentration of 10% and the samples were incubated overnight at 4°C. Supernatant were then removed by centrifugation at 8,000 × *g* for 40 min at 4°C. Pellets were washed four times with 5 ml cold 80% acetone supplemented with 20% Tris-HCl 100 mM, pH 8, and centrifuged at 8,000 × *g* for 30 min at 4°C. Protein pellets were dried for 1 h at 28°C and suspended in Laëmmli sample buffer with β-mercaptoethanol before loading. Proteins were separated by SDS-PAGE using a 6.5% concentrating gel and a 15% separating gel and detected by ultrafast Coomassie staining (Instant*Blue*, Thermo Fisher Scientific). Triplicate experiments were performed to ensure the accuracy of the results.

### Statistical analysis.

GraphPad Prism 8 software (San Diego, CA, USA) was used for statistical analysis. Data were analyzed by nonparametric Mann-Whitney U test (two-tailed) or nonparametric one sample Wilcoxon test (two-tailed). Both significant and nonsignificant exact *P* values were systematically provided. Data are represented as the mean (±standard deviation [SD]) of three independent experiments, each performed in duplicate unless otherwise specified. Error bars indicate SDs.

### Data availability.

The sequence confirming the mini-Tn*5* in both 3H5 and 3H8 mutant strains occurs in a gene assigned to *trpE* can be found at GenBank accession number MT542516.
