# Supplementary material for: Bacterial Long-Range Warfare: Aerial Killing of Legionella pneumophila by Pseudomonas fluorescens
Source: Microbiol Spectr. 2021 Aug 11;9(1):10.1128/spectrum.00404-21. doi: 10.1128/spectrum.00404-21 (PMC8552673; doi:10.1128/spectrum.00404-21)
Supplement: SUPPLEMENTAL FILE 1 — Supplemental material. Download SPECTRUM00404-21_Supp_1_seq13.pdf, PDF file, 0.7 MB [file spectrum00404-21_supp_1_seq13.pdf]

Supplementary Material

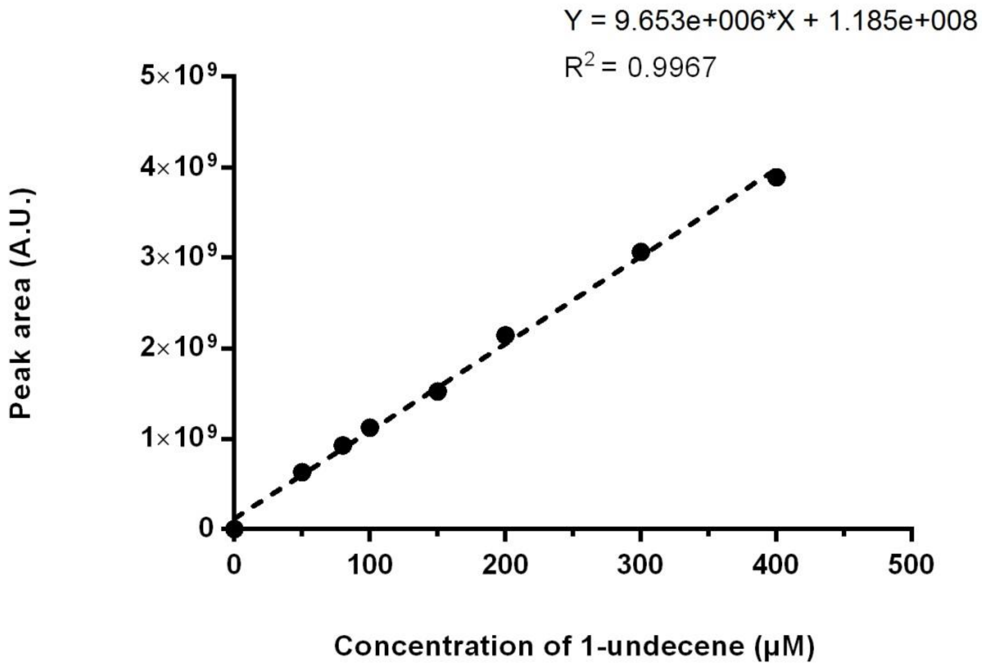

FIG S1 Calibration curve of 1-undecene by GC-MS. A 1 mM stock solution was prepared by diluting pure 1-undecene (97%) purchased from Sigma-Aldrich in absolute ethanol. The calibration points were prepared in a final volume of 100 µl at the following concentrations: 400, 300, 200, 150, 100, 80 and 50 µM. All dilutions were made in absolute ethanol. Samples were then injected (2 µl) and analyzed by GC-MS as described in the Material and Methods section. A.U. indicates arbitrary units.

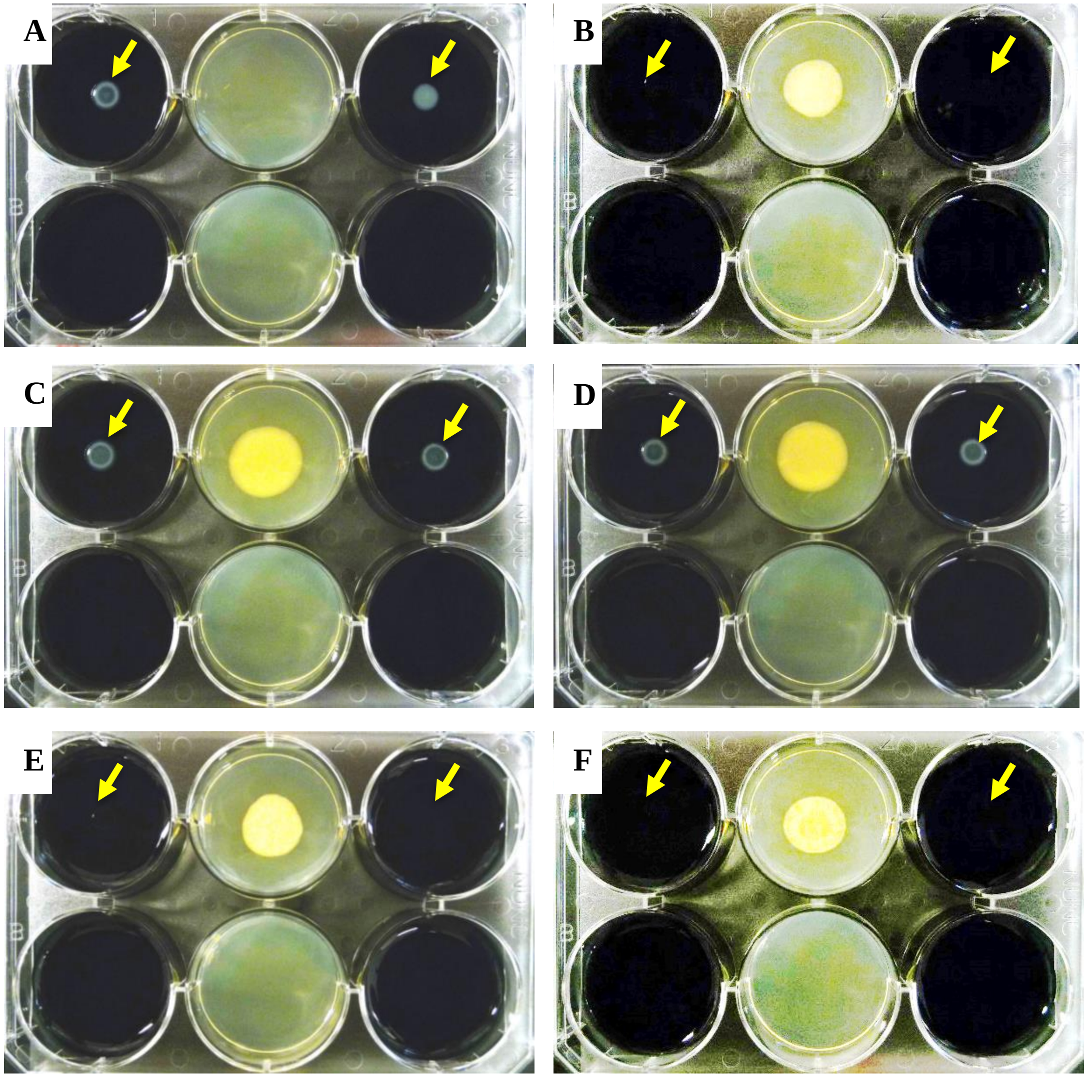

FIG S2 Volatile interference between *P. fluorescens* MFE01, 3H5, 3H5+*trpE*, MFE01Δ*trpE* or MFE01Δ*trpE*+*trpE* and GFP-tagged *L. pneumophila* Lens. A 6-well plate was used to physically separate *L. pneumophila* Lens and *P. fluorescens* MFE01 strains. Each external well was filled with 5 ml of BCYE and internal wells were filled with 5 ml of LB supplemented or not with gentamycin and IPTG according to the strain. Ten µl of a 72 h old culture of *L. pneumophila* Lens and adjusted to a final OD_{600nm} of 0.1 were spotted onto both upper sides of the plate. Finally, 40 µl of a 24 h old culture selected isolate (adjusted to a final OD_{600nm} of 1) were spotted onto the upper centre of the plate. Plates were then incubated at 28°C for 96 h. (A) Control without MFE01. (B) Presence of MFE01. (C) Presence of the 3H5 mutant. (D) Presence of the complemented 3H5+*trpE* strain. (E) Presence of the MFE01Δ*trpE* mutant. (F) Presence of the complemented MFE01Δ*trpE*+*trpE* strain. Images are representative of more than three independent experiments. The initial plating of the bacterial suspension on the agar plate is indicated by yellow arrows.

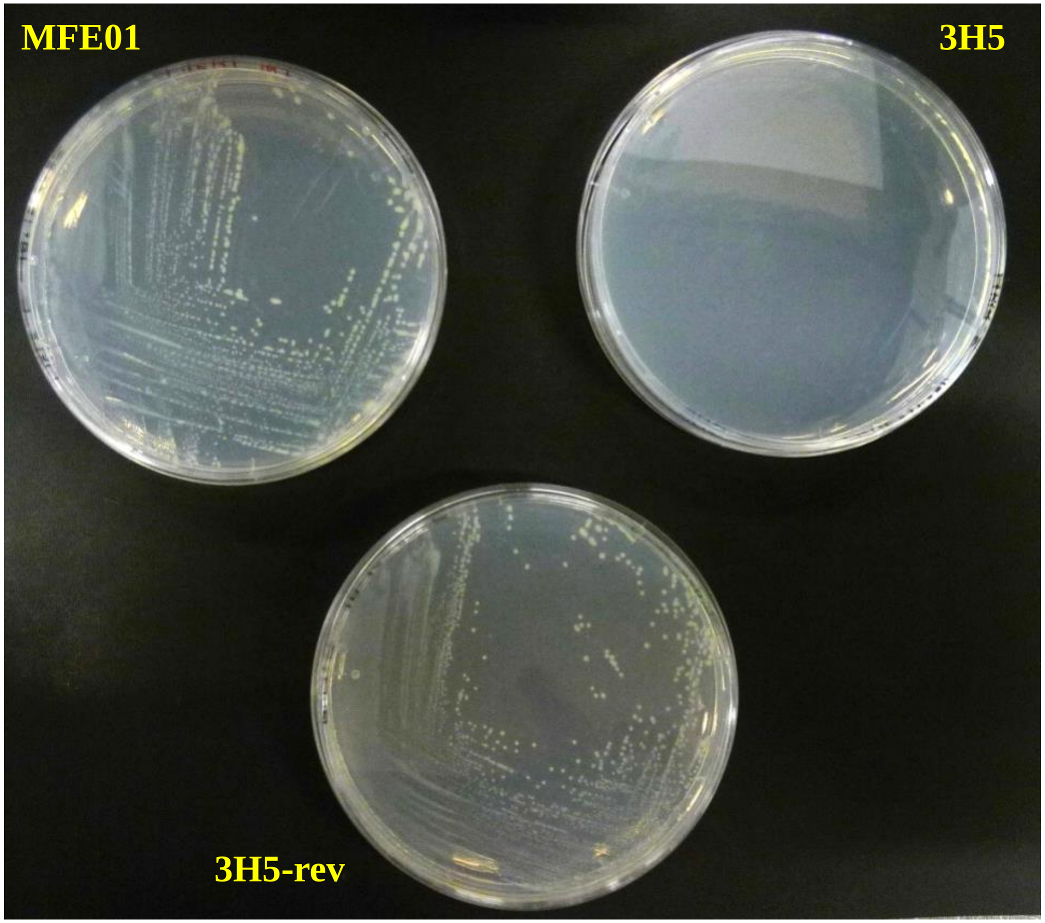

FIG S3 Determination of tryptophan autotrophy in *P. fluorescens* 3H5-rev. Bacteria were grown overnight in LB, washed in MOPS buffer and used to inoculate MOPS Petri dishes (without tryptophan supplementation). Suspensions of 10 µl (OD_{600nm} = 1) were seeded and spread. Petri dishes were incubated at 28°C for 24 h and the presence of colonies indicates that the strains are autotrophic for tryptophan.

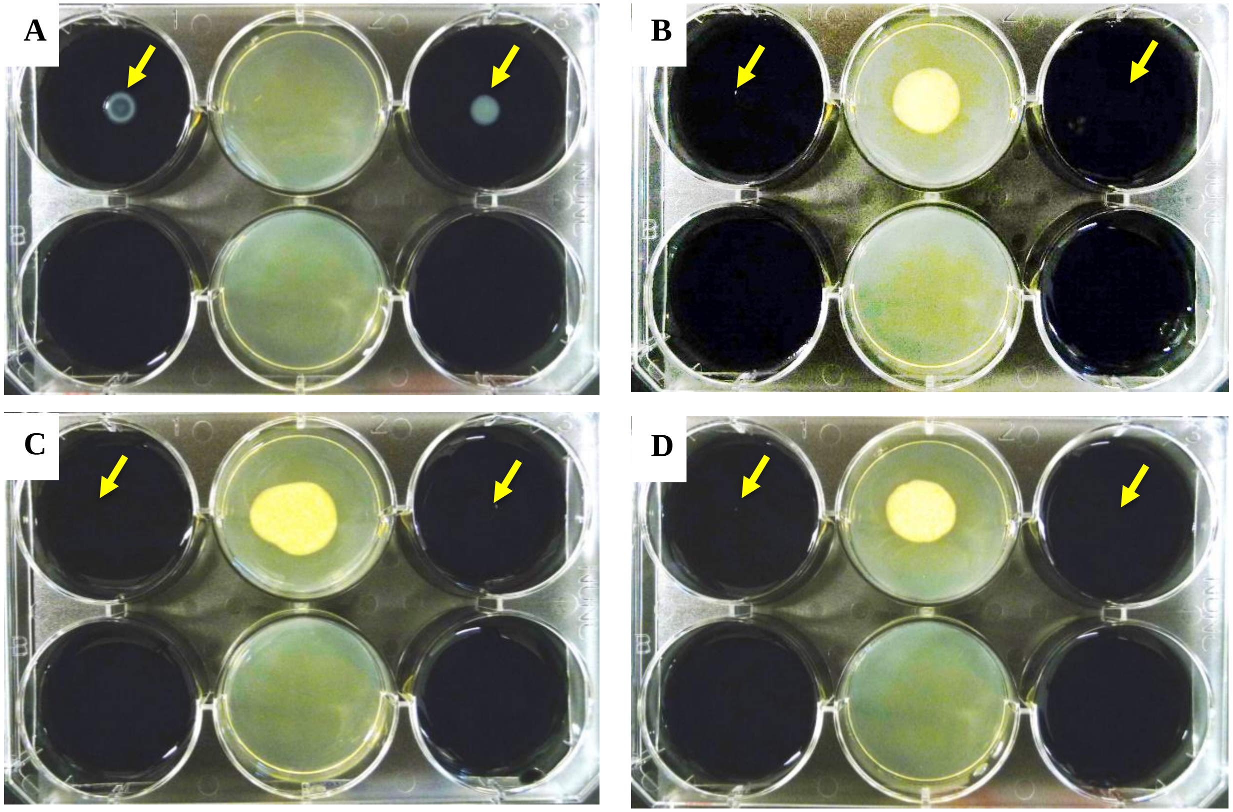

FIG S4 Volatile interference between *P. fluorescens* MFE01Δ*tssC* or MFE01Δ*tssC*-rev and GFP-tagged *L. pneumophila* Lens. A 6-well plate was used to physically separate a *L. pneumophila* Lens and *P. fluorescens* MFE01 strains. Each external well was filled with 5 ml of BCYE and internal wells were filled with 5 ml of LB supplemented or not with gentamycin and IPTG according to the strain. Then, 10 µl of a 72 h old culture of *L. pneumophila* Lens and adjusted to a final OD_{600nm} of 0.1 were spotted onto both upper sides of the plate. Finally, 40 µl of a 24 h old culture selected isolate (adjusted to a final OD_{600nm} of 1) were spotted onto the upper centre of the plate. Plates were then incubated at 28°C for 96 h. (A) Control without MFE01. (B) Presence of MFE01. (C) Presence of the MFE01Δ*tssC* mutant. (D) Presence of the MFE01Δ*tssC*-rev strain. Images are representative of more than three independent experiments. The initial plating of the bacterial suspension on the agar plate is indicated by yellow arrows.

TABLE S1 Strains and plasmids used in this study.

**Strains**	**Relevant characteristics**	**Reference**
P. fluorescens		
MFE01 WT	Air isolate, Rif^R	(1)
MFE01 3A3	Mini-Tn*5* mutant of MFE01	This study
MFE01 3H2	Mini-Tn*5* mutant of MFE01	This study
MFE01 3G4	Mini-Tn*5* mutant of MFE01	This study
MFE01 4G2	Mini-Tn*5* mutant of MFE01	This study
MFE01 3H8	Mini-Tn*5* mutant of MFE01	This study
MFE01 3H5	Mini-Tn*5* mutant of MFE01, *trpE*::Tn*5*	This study
MFE01 3H5+*trpE*	MFE01 3H5 with pPSV35 containing *trpE*	This study
MFE01 3H5-rev	MFE01 3H5 revertant (chromosomal re-introduction of *trpE* at the transposon site)	This study
MFE01Δ*trpE*	MFE01 with *trp*E disruption	This study
MFE01Δ*trpE*+*trpE*	MFE01Δ*trpE* with pPSV35 containing *trpE*	This study
MFE01Δ*tssC*	MFE01 with *tssC* disruption	(2)
MFE01Δ*tssC-rev*	MFE01Δ*tssC* revertant	(2)
L. pneumophila		
CIP 108286	Virulent *L. pneumophila* serogroup 1, strain Lens, Cm^R, *gfp*	(3)
E. coli		
S17-1	RP4-2-Tc::Mu, *aph*::Tn7, *recA*, Sm^R, donor strain for conjugation	(4)
Top10®	F-, *mcrA*, Δ(*araleu*)7697, Δ(*mrr-hsdRMS-mcrBC*), Φ80*lacZΔM15*, Δ*lacX74*, *recA1*, *araD139*, *galU*, *galK*, *rpsL*, Str^R, *endA1*, *nupG*	ThermoFischer Scientific
**Plasmids**		
pPSV35	*P. aeruginosa oriV*, *lacIq*, *mob+*, P*lac*UV5, pUC18 MCS, expression vector, Gm^R	(5)
pPSV35-*trpE*	pPSV35 containing *trpE* from MFE01	This study
pAG408 (ATCC® 87653™)	suicide mini-Tn*5* transposon delivery plasmid	(6)
pAKE604	Ap^R, Km^R, oriT *lacZ*, *sacB*	(7)
pAKE604Δ*trpE*	pAKE604 with deleted *trpE* from MFE01	This study
pAKE604-*trpE*	pAKE604 containing *trpE* from MFE01	This study

TABLE S2 Primers used in this study.

**Primer name**	**Primer sequence (5'→3')**
AV21	GACTCTCCCTTCTCGAATCGTAACCGTTCGTACGA GAATCGCTGTCCTCTCCTTC
AV22	GAAGGAGAGGACGCTGTCTGTCGAAGGTAAGGAA CGGACGAGAGAAGGGAGAG
AV24	CGAATCGTAACCGTTCGTACGAGAATCGCT
I-seq	TACGTGCAAGCAGATTACGG
O-seq	TTGTGCCCATTAACATCACC
Seq-pPSV35-F	TTTACACTTTATGCTTCCGG
Seq-pPSV35-R	AAGGCGATTAAGTTGGGTAA
Muta1-AnthrSyn-F	ATCCAGGGCAAGTACAAC
Muta2-AnthrSyn-*Xba*I-R	TAATAATCTAGAATGGAAATCATCGACGAACT
Muta3-AnthrSyn-*Xba*I-F	TAATAATCTAGAGAAGGTTTCAACGAAGGC
Muta4-AnthrSyn-R	GCTACTTCAAGTGGATCATC
AnthrSyn-*Sac*I-F	TAATAAGAGCTCTATGAATCGCGAAGAATTCC
AnthrSyn-*Xba*I-R	TAATAATCTAGAGAAATTGCGTGGATCAGG
RT-qPCR-*recA*-F	AAGGGTGCCGTAATGCGTAT
RT-qPCR-*recA*-R	ATATCCAGACCCAGAGAGCCAGTA
RT-qPCR-*undA*-F	ATCAAGGACTGCAGCGAGAG
RT-qPCR-*undA*-R	GTACTGGCGCATGGTTTTCG

Reference list

1. Decoin V, Barbey C, Bergeau D, Latour X, Feuilloley MGJ, Orange N, Merieau A. 2014. A type VI secretion system is involved in *Pseudomonas fluorescens* bacterial competition. PLoS One 9:e89411.
2. Gallique M, Decoin V, Barbey C, Rosay T, Feuilloley MGJ, Orange N, Merieau A. 2017. Contribution of the *Pseudomonas fluorescens* MFE01 type VI secretion system to biofilm formation. PLoS One 12:e0170770.
3. Bigot R, Bertaux J, Frere J, Berjeaud JM. 2013. Intra-Amoeba Multiplication Induces Chemotaxis and Biofilm Colonization and Formation for *Legionella*. PLoS One 8:e77875.
4. Simon R, Priefer U, Pühler A. 1983. A Broad Host Range Mobilization System for In Vivo Genetic Engineering: Transposon Mutagenesis in Gram Negative Bacteria. Nat Biotechnol 1:784–791.
5. Rietsch A, Vallet-Gely I, Dove SL, Mekalanos JJ. 2005. ExsE, a secreted regulator of type III secretion genes in *Pseudomonas aeruginosa*. Proc Natl Acad Sci USA 102:8006–8011.
6. Suarez A, Güttler A, Strätz M, Staendner LH, Timmis KN, Guzmán CA. 1997. Green fluorescent protein-based reporter systems for genetic analysis of bacteria including monocopy applications. Gene 196:69–74.
7. El-Sayed AK, Hothersall J, Thomas CM. 2001. Quorum-sensing-dependent regulation of biosynthesis of the polyketide antiobiotic mupirocin in *Pseudomonas fluorescens* NCIMB 10586. Microbiology 147:2127–2139.
